# Efficient sentinel surveillance strategies for preventing epidemics on networks

**DOI:** 10.1371/journal.pcbi.1007517

**Published:** 2019-11-25

**Authors:** Ewan Colman, Petter Holme, Hiroki Sayama, Carlos Gershenson

**Affiliations:** 1 Centro de Ciencias de la Complejidad, Universidad Nacional Autónoma de México, CDMX, Mexico; 2 Tokyo Tech World Research Hub Initiative (WRHI), Institute of Innovative Research, Tokyo Institute of Technology, Japan; 3 Center for Collective Dynamics of Complex Systems, State University of New York at Binghamton, Binghamton, New York, United States of America; 4 Waseda Innovation Lab, Waseda University, Tokyo, Japan; 5 Instituto de Investigaciones en Matemáticas Aplicadas y en Sistemas, Universidad Nacional Autónoma de México, CDMX, Mexico; 6 ITMO University, St. Petersburg, Russian Federation; Institute for Scientific Interchange, ITALY

## Abstract

Surveillance plays a crucial role in preventing emerging infectious diseases from becoming epidemic. In circumstances where it is possible to monitor the infection status of certain people, transport hubs, or hospitals, early detection of the disease allows interventions to be implemented before most of the damage can occur, or at least its impact can be mitigated. This paper addresses the question of which nodes we should select in a network of individuals susceptible to some infectious disease in order to minimize the number of casualties. By simulating disease outbreaks on a collection of empirical and synthetic networks we show that the best strategy depends on topological characteristics of the network. For highly modular or spatially embedded networks it is better to place the sentinels on nodes distributed across different regions. However, if the degree heterogeneity is high, then a strategy that targets network hubs is preferred. We further consider the consequences of having an incomplete sample of the network and demonstrate that the value of new information diminishes as more data is collected. Finally we find further marginal improvements using two heuristics informed by known results in graph theory that exploit the fragmented structure of sparse network data.

## Author summary

In a network of individuals susceptible to some infectious disease, what are the best locations to monitor in order to detect the infection before most damage can be done? In this paper we address this question by considering various heuristic strategies for sentinel placement that can potentially be implemented in real-world situations without requiring excessive amounts of computation, or even having perfect data about the structure of the network. We find that strategies that attempt to distribute sentinels over different regions of the network perform best in highly modular or spatially embedded networks, whereas the strategy of targeting the most well connected individuals works best when there is a considerable amount of contact heterogeneity between individuals. Our results may be used as a guideline to help decide when certain strategies should, or should not, be implemented.

## Introduction

Preventing epidemics is one of the major challenges in public health. In the effort to limit the damage caused by infectious diseases, governments often have to resort to costly vaccination schemes or suffer the human and economic consequences of implementing quarantine programmes. Naturally, there is much to be gained from initiatives that detect outbreaks during their early stages as it allows public health officials to locally contain the spread and prevent it from reaching the wider population. Such *sentinel surveillance* schemes may involve the detection of influenza in airports [1], receiving data from specially selected healthcare centres [2, 3], or monitoring users of intravenously taken drugs [4]. Given that in most situations of this type it is only possible to monitor a fraction of the population at risk, deciding exactly *which* individuals to target is a question that could have significant economic and public health benefits.

This question is similar to the problem of disease control through immunization. A significant literature already exists addressing this problem through mathematical and computational modelling, with the main objective to find the *herd immunity threshold*; defined as the proportion of the population one would need to immunize to ensure that local outbreaks do not develop into epidemics [5]. Much of this work is concerned with the networks of potential transmission pathways for the infectious diseases within a population [6, 7]. Since well connected nodes in such networks are both more likely to receive the infection, and to pass it on to others once they become infectious, it is prudent to locate these nodes and vaccinate them [8, 9]. Various heuristic approaches to find such nodes have been shown to be cost-effective [10, 11].

Unlike targeted vaccination, which remains a theoretical problem, sentinel surveillance is in active use, and, while vaccination strategies on networks have been studied in some depth, much less is known about sentinel surveillance. Moreover, recent results show the best candidates for vaccination are not necessarily the same as those for sentinel placement [12]. While methods have been developed to find the optimal placement of *n* sentinels on a given network whose structure is known [13–15], fewer studies consider heuristic approaches that do not require perfect data [16]. While incomplete or unreliable information has been investigated in the context of influence maximization and other centrality measures [17, 18], questions still remain about sentinel placement in the disease context.

The premise of our investigation is that a good strategy is one that selects nodes that have many connections but are also not too close to each other. While previous work in this area has focused on locating well connected individuals [19–21], here we ask whether a strategy that also distributes the sentinels across different regions of the network can be better than one that simply targets the highest degree nodes.

The first part of this paper addresses the question of how network topology affects the performance of different strategies for sentinel placement. We generate networks with varying amounts of degree heterogeneity as well as varying assortativity, in one case, and spatial structure in another. We simulate the spread of disease on these networks and introduce three sentinel placement strategies that utilize different aspects of the network topology. We then present results for a wide range parameter combinations to show that the best strategy for a given network depends on its topology.

The second part of this paper deals with the issue of incomplete data. We introduce three sentinel placement strategies and show that, in addition to the network structure, the performance depends also on the size of the sample. We discuss how the value of additional data decreases as the sample size increases. We finally explore the hypothesis that a known result from random graph theory can be used as a guide to help decide which strategy to employ.

## Methods

### Network topology and sentinel performance

We start from the intuitive hypothesis that nodes with the largest degree are the best candidates for placing sentinels since these nodes are typically the most likely to receive and propagate the disease. We will compare this idea to strategies that distribute the sentinels in such a way that each one covers a different region of the network. We test each strategy on a range of networks with different levels of degree heterogeneity, and different levels of either spatial or modular structure. We start by describing how these networks were generated and then describe the process of simulating epidemics on the networks. We then explore the consequences of implementing three strategies for sentinel placement and introduce two measures of efficacy for a sentinel placement based on the results of disease simulations.

#### Generating synthetic networks

Our approach to generating networks with tunable degree heterogeneity and group assortativity (or spatial structure) is a modified version of the *configuration model* [22]. In the basic model, *N* nodes are considered, and each node, *i*, is given a degree, *k*_*i*_. We can think of the node *i* as having *k*_*i*_ adjacent half edges (or stubs) attached to it. Each stub is then paired with another stub to create an edge. The pairs are randomly selected with the following conditions: (a) that the two stubs must belong to different nodes (no self-loops), and (b) the stubs must belong to pair of nodes that are not already connected (no multi-edges).

The goal is to create a degree distribution with a specified mean degree *μ*, and standard deviation *σ*, which will be our measure of degree heterogeneity. To achieve this we first assign *μ* stubs to every node (*k*_*i*_ = *μ* for all *i*). We then begin a procedure of preferential rewiring: in each iteration two nodes, *i* and *j*, are selected. The first (*i*) is chosen randomly from all nodes that have degree greater than 1, and the second (*j*) is selected with probability proportional to its degree. A stub is then removed from the first node (*k*_*i*_ :→ *k*_*i*_ − 1) and attached to the second (*k*_*j*_ :→ *k*_*j*_ + 1). This process is repeated until the standard deviation of the node degrees is larger than the specified value *σ*. The degree distributions generated through this process are compared with those of real networks described in the *Data* section.

We consider two classes of modified configuration model. These are:

**Modular**: We create *m* modules, each consisting of *n* nodes. Every stub is chosen to be either *intra-module*, with probability *p* (the *module assortativity*), or *inter-module*, with probability 1 − *p*. Intra-module stubs can only be paired with stubs belonging to nodes of the same module, inter-module stubs can only be paired with other inter-module stubs.
**Spatial**: Nodes are placed on a circle evenly spaced with distance of 1 between them (around the circumference). Two stubs can only be paired together if they are withing a distance *r* (the *connection radius*) of each other.

**Fig 1:**
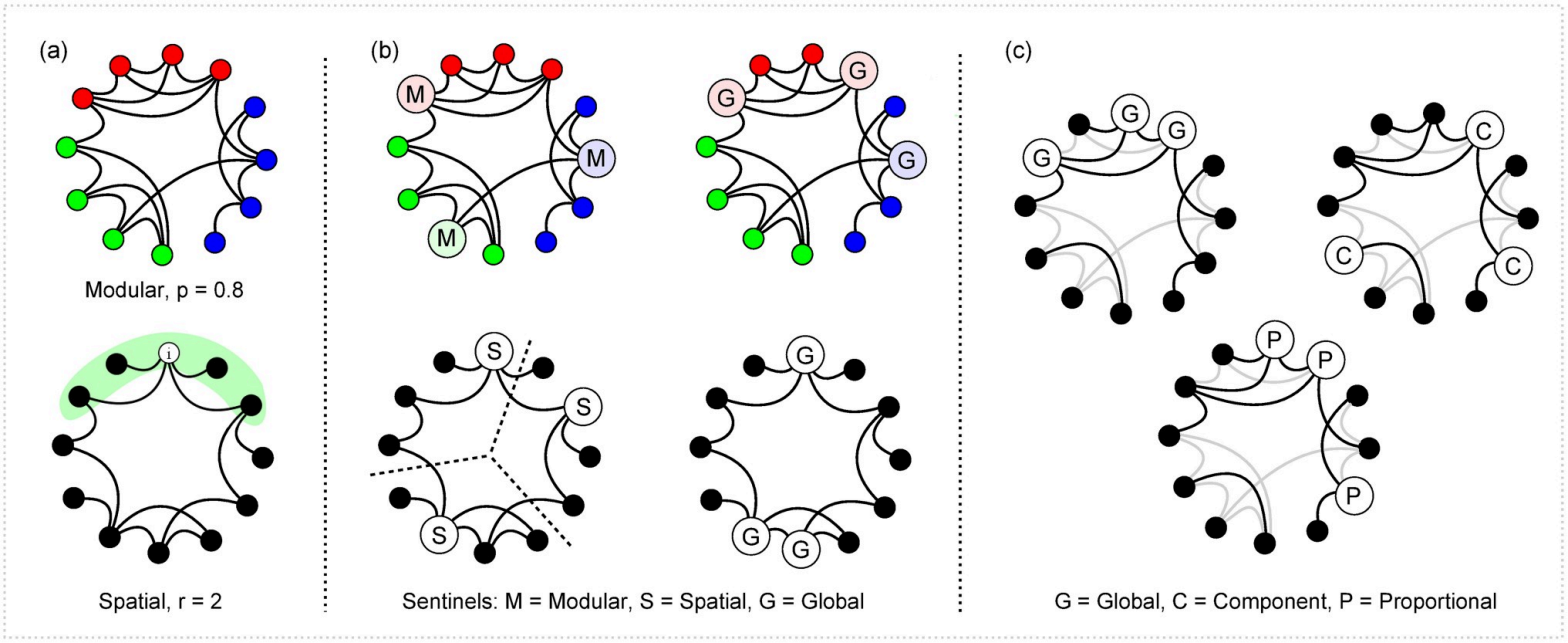
Network generation and sentinel strategies. (a) Examples of the two types of network. In the modular network *p* represents the probability that an edge will connect 2 nodes of the same colour. In the spatial network, edges can only connect nodes that are within distance *r* of each other e.g the edges of *i* are all in the green region. (b) Examples of sentinel placement strategies. (c) Strategies for networks with limited data. Examples of sentinel placement strategies with s = 3.

Examples are shown in Fig 1(a).

#### Disease model

We use a Susceptible-Infected-Recovered (SIR) model of disease propagation. Initially the entire population is in the susceptible state except for one randomly selected seed node that is in the infectious state. Nodes that are in the infectious state infect their susceptible neighbours at a rate of *β* per unit of time. When this occurs the susceptible neighbour will transition to the infectious state for a given infectious period duration before transitioning to the recovered state. We consider two possible ways to terminate the simulation:

**Single seed**: The simulation ends when all individuals are in either the susceptible or recovered state.
**Multiple seed**: After all individuals infected directly or indirectly from the initial seed are in the recovered state, a new seed is randomly selected from the remaining susceptible population and made to transition to the infectious state, thus allowing disease propagation to continue between the remaining susceptible nodes. This process repeats until the entire population is in the recovered state.

The multiple-seed simulation corresponds to diseases that remain hidden as they spread through a population; without the aid of sentinel surveillance they will reoccur indefinitely. The single-seed version, on the other hand, is more appropriate for diseases that result in the infected individual very quickly going to hospital where the disease will be diagnosed (and thus detected). Note that for the multiple-seed simulation it is not required that we specify the amount of time between the end of one outbreak and the beginning of the next.

For our analysis here we use an infectious period of duration 1 (without loss of generality since there are no units of time imposed) and transmission rate *β* = 0.5. For each combination of parameter values tested, we generated 10^3^ networks and ran 10^2^ disease outbreaks on each one. The results presented are the means over all 10^5^ simulated outbreaks. Simulations were performed using the SIR code from the Epidemics on Networks (EoN) Python library [23].

#### Sentinel placement strategies

We consider the following strategies for deciding the placement of *s* sentinels:

**Spatial**: Divide the network into *s* spatial regions of equal size. Place a sentinel on the highest degree node in each region.
**Modular**: Place a sentinel on the highest degree node in each of *s* different modules
**Global**: Place sentinels on the *s* highest degree nodes.

Examples of each of these are shown in Fig 1(b). We will apply the spatial and modular strategies to the spatial and modular networks, respectively, and the global strategy to both.

#### Measuring strategy performance

We use two complementary approaches to measuring the performance of a sentinel placement strategy. The first measure is applied to the single-seed disease simulations and counts the proportion of cases in an outbreak that could potentially have been prevented as a consequence of being detected. Specifically, we measure the size of the full outbreak and subtract from this the number of cases that occurred before the outbreak reached any of the sentinels. If no sentinel receives the disease then we subtract all the cases that occurred, giving a result of zero. Formally, for a set of sentinels *S*, we define the *cases after detection*, Φ_*A*_(*S*), for a given outbreak as

$$\begin{array}{c} \Phi_{A}\left(S\right)=I\left(\infty \right)-I\left(\underset{s\in S}{\text{min}}\left(\text{infection}\;\text{time}\;\text{of}\;\text{s}\right)\right) \end{array}$$ (1)

where *I*(*t*) is the number of cases (infected or recovered) at time *t*. Note that this only measures the number of cases that could *potentially* be prevented and we do not consider any of the difficulties of actually preventing them nor do we consider the time it would take to implement any such intervention.

The second measure is applied to the multiple-seed disease simulation outcomes and counts the proportion of cases that occurred before the outbreak was detected, i.e. before it reached any of the sentinels.

$$\begin{array}{c} \Phi_{B}\left(S\right)=I\left(\underset{s\in S}{\text{min}}\left(\text{infection}\;\text{time}\;\text{of}\;\text{s}\right)\right) \end{array}$$ (2)

We have chosen two measures to give different results that complement each other. One drawback of the first measure is that when outbreak goes undetected we have Φ_*A*_ = 0. Hence, the mean of Φ_*A*_ over many simulations is deceptively small in sparse or fragmented networks where outbreaks tend to be very small. On the other hand, Φ_*B*_ takes into account the small outbreaks, but the assumption that the disease will reappear in the population (after an unspecified amount of time) is unrealistic for diseases with considerable symptomatic burden that are likely to reveal themselves in other ways. A range of alternative measures has been considered in [24].

### Sentinel placement with incomplete data

The objective of this section is to test the performance of sentinel placement strategies given limited information about the network structure. We use both empirical and synthetic network data to ask how well different strategies will perform when only a sample of the edges are known.

#### Sampling regime

We consider two types of sampling. For a give percentage *X*

**Edge**: Select a random sample of *X*% of the edges from the true network and include all nodes attached to at least one of these edges.
**Node**: Select an initial random sample of *X*% of the nodes from the true network and include all edges and additional nodes adjacent to at least one node in the initial sample.

Edge sampling is typically used when the available information is in the form of an interaction. For example, flights between airports, messages sent on social media and, potentially, proximity interactions recorded through mobile phones. Node sampling occurs when survey respondents are asked to name the people that they have interacted with; this is more typical for the hidden communities of, for example, drug users.

In both cases we expect that an incomplete sample network would be fragmented into a number of disconnected components, where a *component* is defined as a set of nodes which are connected through a sequence of edges. Here we introduce strategies that take advantage of this fragmentation by exploiting the fact that different components are likely to belong to different regions of the complete network such as modules or spatial areas.

#### Sentinel placement strategies

Suppose we have a sample of *X*% of the edges (or nodes) of the network. We use *C*_*i*_ to denote the size of the *i*th largest component and *N* to be the total number of nodes in the full network. We consider the following strategies:

**Component**: Choose the highest degree node in each of the *s* largest components. If the number of components is less than *s*, cycle through the components in reverse order of size, choosing the best available node each time, until *s* sentinels have been selected.
**Proportional**: Choose the ⌈*sC*_1_/*N*⌉ highest degree nodes from the largest component, then ⌈*sC*_2_/*N*⌉ from the second largest component and so on until *s* sentinels have been selected.
**Global**: Choose the *s* nodes with highest degree in the sample.

In all three cases we assume that all nodes in the network are known regardless of whether they are connected to an edge or not (thus at small sample sizes the sample network will include a large number of nodes with degree 0). Examples of these strategies are shown in Fig 1(c).

#### Data

We obtained 6 freely available datasets from the Sociopatterns project. This data was collected by providing participants with RFID technology that logged every instance in which two participants were within a short distance of each other. The settings in which these experiments were conducted are a conference [25], a hospital [26], a primary school [27], a high school [28], an office workplace [29], and a collection of households in rural Kenya [30]. A precise description of each experiment can be found in these references. In each case the data included contacts between pairs of participants and the time that the contact happened. From these data we constructed unweighted static (time-aggregated) networks in which nodes are participants and edges exist between any pair of participants who shared at least one contact.

In addition, we used 3 transport networks constructed from timetable data for all of the United Kingdom. Details of this dataset are found in [31]. From these data we use the network of airports and domestic flight paths, the network of railway stations and lines between them, and the London underground metro system, which we found by taking the largest connected component of the whole UK metro network.

Furthermore, we obtained 8 networks created from surveys of individuals thought to be at risk of becoming infected with diseases through sexual or drug-taking contact [32]. A fraction of the participants in these studies were found through healthcare clinics while the remainder were found by referral from other participants; a process which is known to introduce biases in the data and is likely to give higher values of degree heterogeneity and lower levels of modularity than would a random sample [33]. Consequently, the majority of the nodes are individuals who were referred by others but did not themselves participate in the survey. To create a network that realistically represents the potential pathways of transmission, we removed all nodes of degree one and took the largest connected component of the remaining network. In one case this component consisted of only a single node and so we omitted this dataset.

In addition to the 16 empirical networks described above, we created 200 synthetic networks using the processes described in the section *generating synthetic networks*. These were generated with a range of randomly selected topological characteristics. The first 100 networks were generated using the modular model with the number of modules chosen uniformly at random between 3 and 8, the module size between 10 and 30, and the assortativity parameter between 0.4 and 1. The second 100 were generated using the spatial model with the number of nodes drawn uniformly between 100 and 200, and the connection radii between 10 and 60. In both the spatial and modular networks, the mean degree was chosen uniformly at random between 4 and 9, and the standard deviation of the degree distribution between 0 and 5.

The degree distributions for a the empirical networks are shown in supplement (S1 Fig). For comparison, the degree distributions for a sample of the synthetic networks are also shown (S2 Fig).

#### Disease model

The disease simulation was performed as described earlier. To adjust for the varying levels of connectivity across different networks we chose *β* = 2/*μ* where *μ* is the mean degree of the particular network. Thus, at the beginning of the outbreak, the randomly selected seed node is expected to infect 2 other individuals. For each network 10^2^ outbreaks were simulated. Distribution of outbreak sizes are shown in S3 Fig. The three strategies were applied to 10^3^ different random samples of each network using the edges sampling method, and again using node sampling. To evaluate the performance of each strategy we again use the same measures as before. The values presented for Φ_*A*_ and Φ_*B*_ are the mean over all 10^5^ combinations of edge sample and simulated outbreak.

## Results

### Network topology and sentinel performance

**Fig 2:**
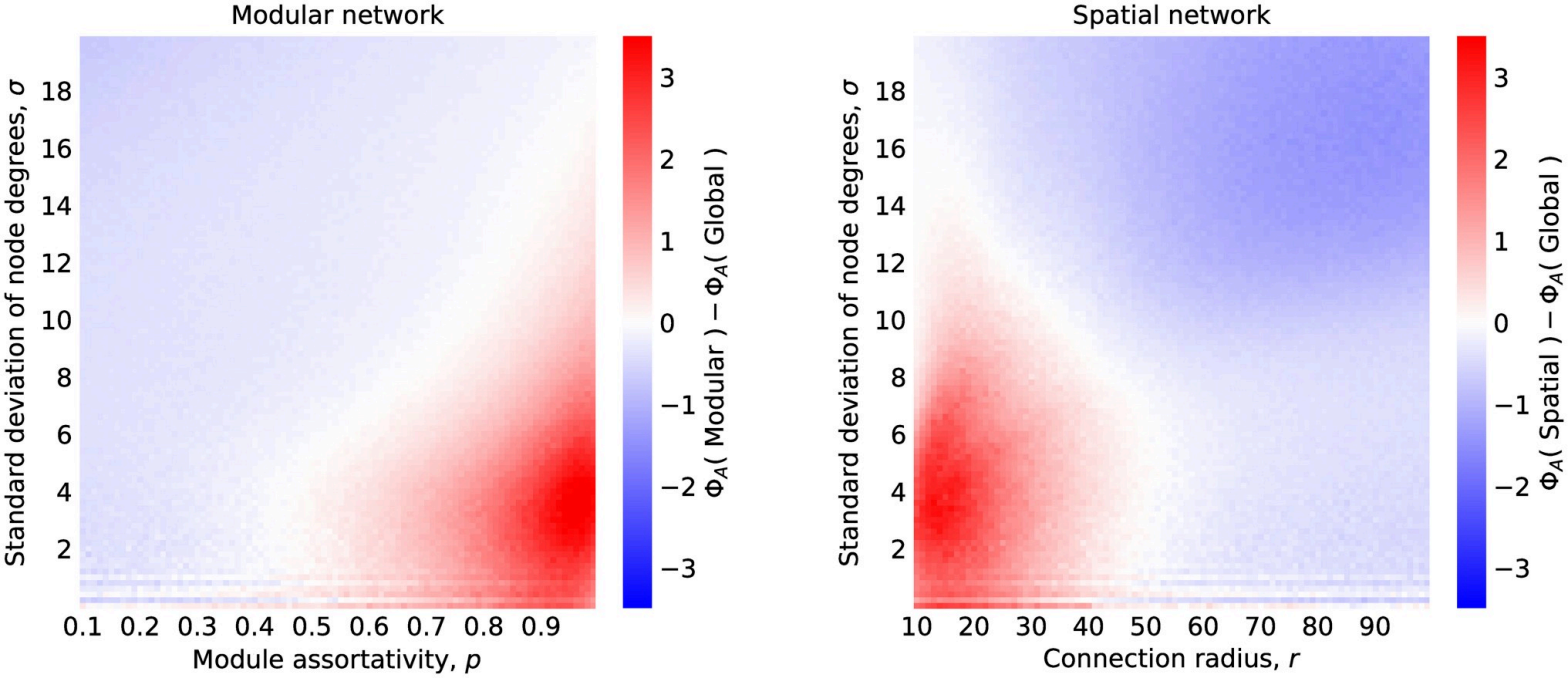
Strategy performance. The difference in effectiveness between the degree based strategy and the strategies based on network subdivision. Blue areas indicate that the global strategy performs better than the modular (left) or spatial (right) strategy. Red areas indicate the opposite.

We performed disease simulations on modular networks with *m* = 5 modules and *n* = 40 nodes per module over a range of values of *p* and *σ*, and on spatial networks with *N* = 200 nodes over a range of values of *r* and *σ*. We measured the performance of the sentinel placement strategies, arbitrarily choosing *s* = 5. Fig 2 shows the difference in the number of cases after detection, Φ_*A*_, between the global strategy and the modular or spatial strategy for their respective type of network over a range of values of degree heterogeneity, module assortativity (in the modular network), and connection radius (in the spatial network).

For the modular networks, when the degree distribution is relatively homogeneous, *σ* ≲ 6, we see that the nodes selected using the global strategy are no better than when *σ* = 0 and the sentinels are essentially random. This is also true for nodes selected by the modular strategy when *p* = 0.5, when edges are equally likely to appear within modules as they are between modules. We see that in disassortative networks, when *p* < 0.5, that the global strategy performs better than the modular strategy, whereas when *p* > 0.5 the opposite is the case. As we look at networks with higher degree heterogeneity *σ* ≳ 6 we see that the global strategy starts to beat the modular strategy even in assortative networks (*p* > 0.5) until eventually, at *σ* ≈ 18, the global strategy dominates for all levels of assortativity.

A similar story can be told for the spatial networks. In networks with homogeneous degree distributions, the spatial strategy is preferred for when the connection radius is small. We have chosen only to plot values of *r* up to 10^2^ since beyond this point edges can potentially appear between any two nodes making the network equivalent to the original configuration model. However, the threshold value for which the spatial strategy is no longer preferred to the global strategy appears at around *r* ≈ 50. Again, the spatial strategy becomes increasingly redundant as we look at networks with larger degree heterogeneity.

### Sentinel placement with incomplete data

#### The effect of sample size on strategy performance

**Fig 3:**
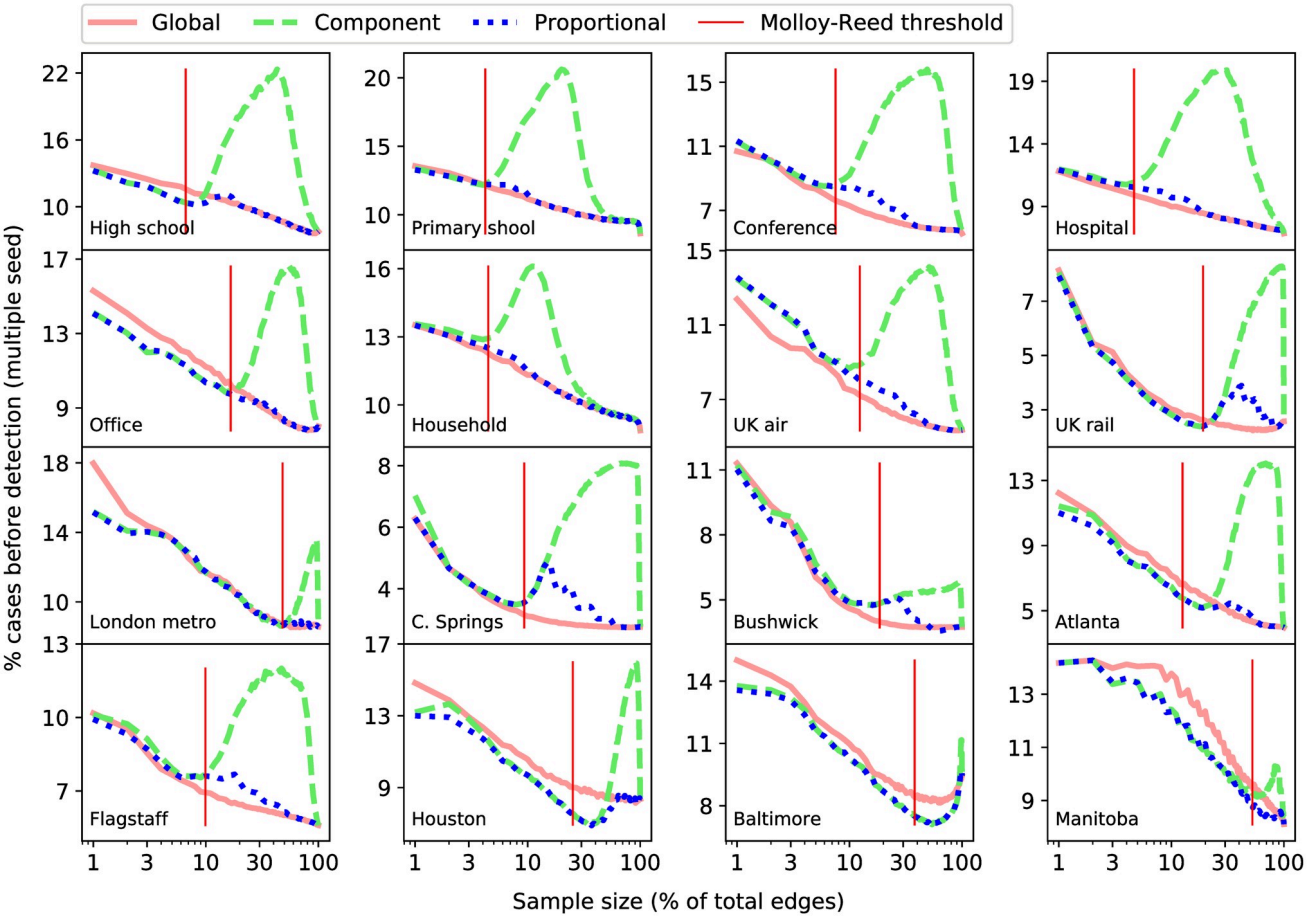
Edge sampling. Strategy performance as a function of the number of edges in the sample under the *edge* sampling regime. Each plot corresponds to a different empirical network. Each line represents a different strategy. Results are given as the mean percentage of the nodes in the network infected before at least one sentinel was infected. The vertical red line corresponds to the threshold value found using the method described in the section *Connection to criticality in the configuration model*.

For each network and each sampling method we would like to know which strategy performs better. Since we are considering samples of edges (or nodes) in the network, we also want to know how the percentage, *X*, of edges (or nodes) that are included in the sample effects this outcome. We start by focusing on the edge sampling regime and explain the relationships observed in Fig 3.

When the sample size is 0, the strategies are equivalent to simply choosing random nodes. When the number of edges is approximately the same as the number of sentinels, all three strategies act in a way that is similar to acquaintance selection, whereby sentinel nodes are selected by following a random edges of a randomly selected node (this method has been demonstrated to be better than a purely random strategy) [34]. As the sample size increases all three of our strategies are able to exploit the additional data to their advantage and performance improves well above these baselines (S4 Fig).

Notably, most of the improvement results from increasing the sample size from 1% to 10%. Beyond 10% the value of information, i.e. the performance gained with each additional edge sampled, is significantly lower. We conclude then, that in many situations it may not be efficient to gather information about the entire network—a relatively small sample may be more cost-effective. The rest of our analysis focuses on achieving additional improvements using strategies that exploit fragmentation.

Focusing on Φ_*B*_(Component) we immediately see that the performance improvement is not monotonic. The reason is as follows: as the sample size approaches a certain value, the small components begin to coalesce into a single large component, leaving many very small components made up mostly of peripheral nodes that typically have very few edges. By design the component strategy will select these peripheral nodes despite it being smarter to choose multiple sentinels from the large component (we discuss this further in the following section). Finally, as the large component begins to account for the entire network, Φ_*B*_(Component) begins to decreases again as it starts to choose multiple sentinels from the large component.

To see how network topology influences the outcomes we computed the maximum modularity, *Q*, of each network using the Louvain method [35] (hereafter referred to simply as modularity), and the normalized heterogeneity of the degree distribution *σ*^2^/*μ* where *μ* and *σ* are the mean and standard deviation of the degree distribution respectively. These values are shown in Table 1. We observe in Fig 3 that the networks for which the component strategy is, at small sample sizes, better than the degree strategy, are those for which modularity is highest. Heterogeneity, on the other hand, does not appear to influence the relative performance of the strategies as strongly, nor do the number of nodes and the number of edges despite their known effects on modularity.

**Table 1:** Datasets and statistics. Degree heterogeneity is represented by the variance of the degree distribution divided by its mean, *σ*^2^/*μ*, and *Q* is the modularity of the network.

Data	Nodes	Edges	*σ*^2^/*μ*	*Q*
Baltimore	558	729	1.03	0.92
UK rail	2490	4387	2.70	0.89
Houston	377	634	1.64	0.84
London metro	307	373	0.51	0.84
Atlanta	340	783	4.44	0.77
High school	326	2139	3.07	0.74
Bushwick	263	366	3.66	0.71
C. Springs	905	2205	6.88	0.66
Primary school	242	2645	3.09	0.63
Office	90	242	1.81	0.60
Manitoba	33	34	0.94	0.60
Flagstaff	165	504	5.12	0.49
Conference	110	478	5.92	0.34
UK air	45	123	4.62	0.32
Household	47	506	2.78	0.25
Hospital	74	609	6.95	0.18

The proportional strategy improves on the component strategy by eliminating the possibility of choosing nodes from very small, peripheral, components. Since it retains the advantages of the component strategy at small sample sizes, we observe that it is always at least as good as the component strategy. Thus, the proportional strategy can be considered to be the best option in cases where modularity is relatively high. In the cases where this strategy outperforms the global strategy, however, there are ranges of sample sizes over which the performance decreases; a situation we would clearly like to avoid. In the following section we introduce a method to approximate the sample size at which this behaviour occurs.

**Fig 4:**
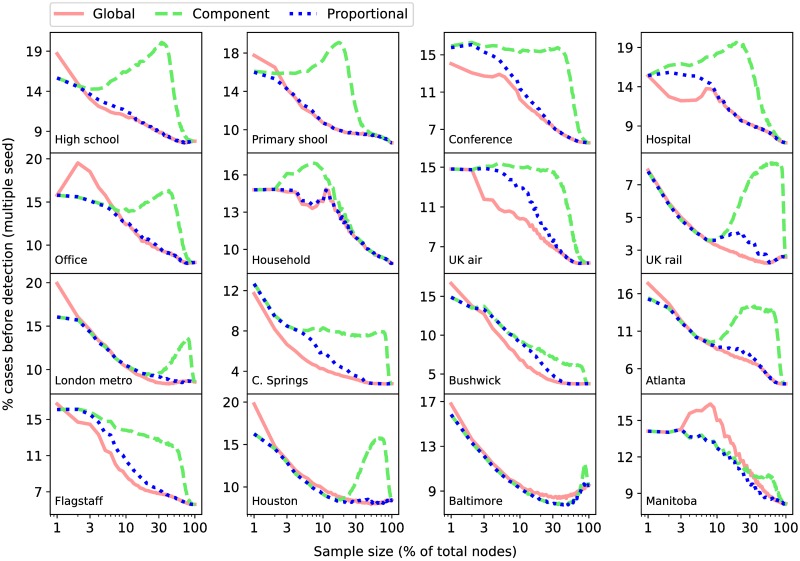
Node sampling. Strategy performance as a function of the number of nodes in the sample under the *node* sampling regime. Each plot corresponds to a different empirical network. Each line represents a different strategy. Results are given as the mean percentage of the nodes in the network infected before at least one sentinel was infected.

The equivalent results for the node sampling regime are shown in Fig 4. At small sample sizes the largest components tend to consist of the highest degree nodes that were sampled directly and all of their neighbours (which all have a degree of 1 in the sample). Thus, in many cases the nodes chosen by the component strategy are the same as those chosen by the global strategy. For slightly larger sample sizes, nodes that have high degree in the sample are likely to be linked, directly or indirectly, to other high degree nodes, leaving one component containing many of the high degree nodes and a few smaller peripheral components containing only small degree nodes. Consequently, node sampling is notably worse than edge sampling for the component strategy.

There are a few cases when node sampling is applied and the performance of the global strategy does not monotonically increase with sample size. One possible reason is as follows: at small sample sizes, the sentinel nodes are those selected as focal nodes during the sampling process (their degree is highest due to the sampling procedure). Hypothetically, a node could be connected to every other node in the network and not be selected as a focal node; if the number of focal nodes selected is smaller than the number of sentinels, then the hub will definitely be selected as a sentinel; if the number of focal nodes is higher, then it is possible that only the focal nodes will become sentinels and the best node, i.e. the hub, is rejected and the performance is worse than it was at a slightly smaller sample size.

We found the same conclusions can be made looking at the other performance measure Φ_*A*_, however, these results are more affected by characteristics of the network data. To test the robustness of our results we repeated the analysis using 3 and 10 sentinels (instead of 5) and found nothing that disagreed with the results as they are presented here. The corresponding figures for both sampling methods, performance measures, and number of sentinels, can be found in S5–S16 Figs.

The important question to ask here is whether the component or proportional strategies should be chosen by policy-makers. Figs 3 and 4 show in some networks at particular ranges of sampling percentages that the global strategy is not the best choice on average. We find, however, that the distribution of values, Φ_*A*_ and Φ_*B*_, over all performed simulations have large standard deviations. Hence, the values shown in these figures do not give a good indication of which strategy would give a better outcome for one particular network sample; as this is all we would expect to have in reality. The following section proposes a way to deal with this issue.

#### Connection to criticality in the configuration model

It appears that the component and proportional strategies perform best when the number of edges is large enough to give information about degrees of nodes, yet not so large that one component dominates the network. Following this, we pose that a result from graph theory that links degree heterogeneity to the percolation threshold in configuration model networks can be used to find this optimum sample size. The particular result, first found by Molloy and Reed [22] and also found through different methods by Newman [36], connects the degree distribution of a network to the emergence of a giant component. Suppose we have a configuration model network with a large number of nodes, and *p*_*k*_ is the probability that a node has degree *k*, then the network has a giant component (defined as one that contains a finite fraction of the nodes in an infinite network) when

$$\begin{array}{c} \sum_{k}k\left(k-2\right)p_{k}=0. \end{array}$$ (3)

This formula connects to our approach to sentinel placement in the following way: for any given sample of edges we can infer a value of *p*_*k*_ by dividing the number of nodes that have degree *k* in the sample by *N*, the total number of nodes. If the sample of the empirical network shows some level of similarly to the configuration model, then it is likely that a dominant component will form when Eq (3) is satisfied. Moreover, we postulate that the sample size for which this is most likely to be true will also be the sample size that yields the best results for the component-based strategies.

We therefore want to know how well the sample size that satisfies Eq (3) predicts the sample size corresponding to the local minimum in Φ_*B*_, using the component strategy. For each network, we first calculated the value of *X* that gives the first minimum in the performance of the component strategy. We then calculated the value of *X* that most closely satisfies Eq (3) (based on the mean over all 10^3^ samples of size *X*).

**Fig 5:**
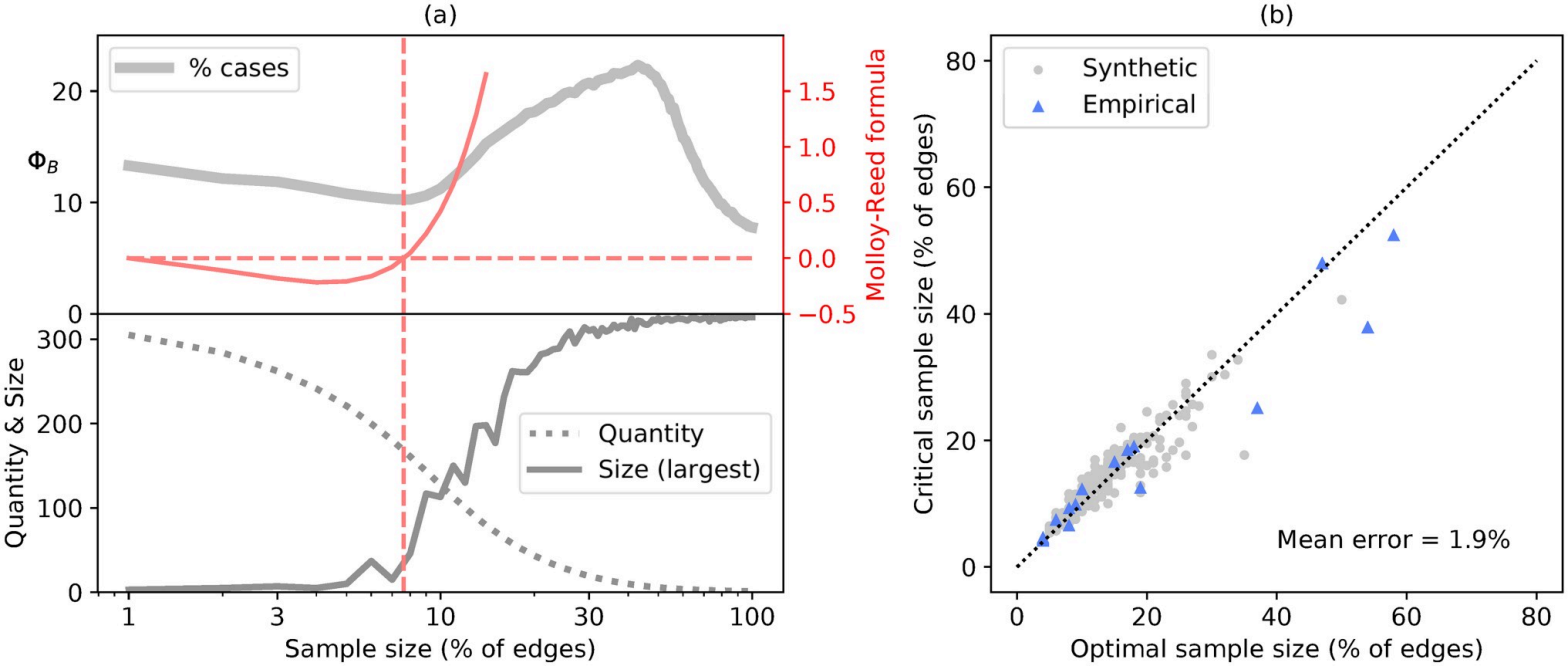
Theory as a predictor of optimal sample size. (a) Upper: The grey line represents the component strategy when applied to the high school network data as seen in Fig 3. The solid red line represents the left hand side of Eq (3) as a function of the sample size. The dashed lines indicate the sample size for which Eq (3) is satisfied. Lower: The size of the largest connected component in the sample and the number of components. (b) Each point represents one network. The dashed line represents where both these values are the same. The optimal value is the location of the first local minimum as we look at increasing sample sizes. The error value presented is the mean absolute error.

We see in Fig 5 that the sample size calculated from Eq (3) corresponds very closely to the sample size that minimizes the number of cases before detection for the component strategy. If one was to use this as a guide for deciding how much data to collect, given that they are using the component or proportional strategy, they will on average be within 2% of the optimum. For the samples generated using the node sampling method we find that this result is not applicable. The increased degree heterogeneity of the sample causes the left hand side of Eq (3) to always be greater than 0.

Our final analysis addresses the question of whether these results relevant or useful in any way. The previous result indicates that situations may occur for which the component strategy would be a preferable choice to the global strategy. While the Global strategy is the best option in the majority of circumstances, we might wish to know if circumstances also exist for which this is not the case. Based on our findings so far, we know that these cases are most likely to occur when the network is sufficiently fragmented (which is indicated by ∑_*k*_ *k*(*k* − 2)*p*_*k*_ ≤ 0), and also when modularity is high and heterogeneity is low.

To demonstrate this we take each individual sample over all sampling percentages and removed all those for which ∑_*k*_ *k*(*k* − 2)*p*_*k*_ > 0. For those remaining *sufficiently fragmented samples* we used both the global and components strategies to find the sentinels. If the sentinels identified were exactly the same using both strategies then we omit these cases. If they are different then we compute the difference in their performance. We find that all the differences computed are significantly different to zero with extremely small p-values (Wilcoxon signed rank test with the Pratt method for dealing with zeros [37]).

**Fig 6:**
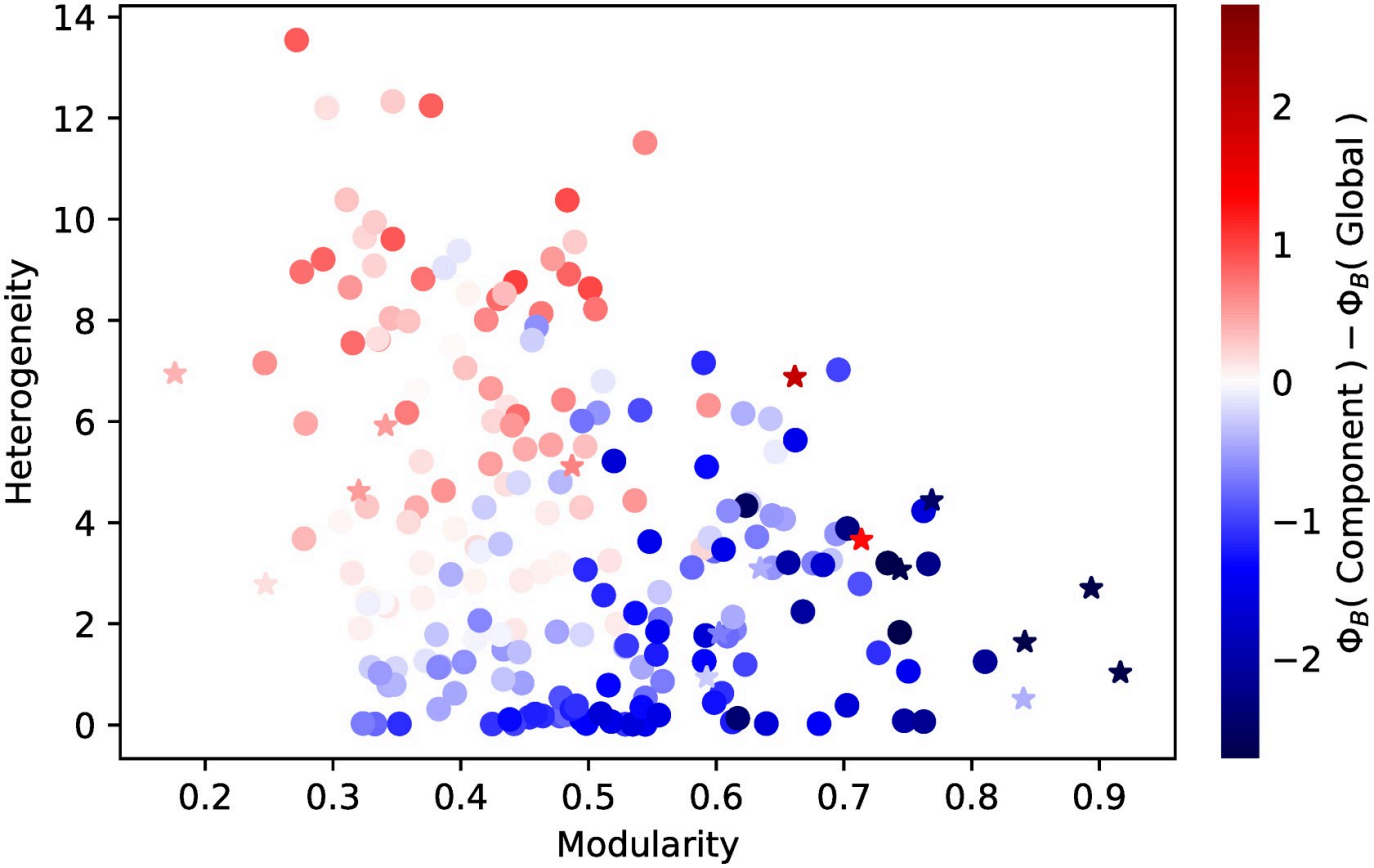
Difference in the number of cases before detection for sufficiently fragmented samples. Each marker represents one of the networks described in the Data section. Empirical networks are represented by star shaped markers, synthetic networks are represented by circles. The edge sampling method was used and only samples that were evaluated to be sufficiently fragmented contribute to the results shown here (the corresponding figure for node sampling is presented in the supplement). Red markers show where the global strategy performs better, i.e. yields a smaller number of cases, than the component strategy.

The mean for Φ_*B*_ is shown in Fig 6 plotted against the heterogeneity and modularity of the underlying network (computed from the full data set, not the sample). We see that there are a considerable number of samples for which the component strategy would be preferred to the global strategy, and these are those for which heterogeneity is low and modularity is high. The corresponding figures for Φ_*A*_ for different sampling methods and numbers of sentinels are presented in S17 and S18 Figs.

## Discussion

There are many obstacles in the effort to prevent future epidemics. Here we have explored just one of these challenges: the question of how to choose susceptible individuals for frequent monitoring in a way that is both effective and cheap. We have demonstrated through simulations on a range of networks, both empirical and synthetic, that network topology plays a significant role in determining the overall efficacy of a sentinel placement strategy. We have asked the question of whether a strategy that distributes sentinels across different regions of a network could outperform one that simply targets the most well connected nodes, and when should either strategy be applied.

We have demonstrated in the section *Network topology and sentinel performance*, the difference that network topology makes to the optimal choice of strategy. We observed that the segregation of nodes into different communities or spatial regions drives the performance of any given strategy, particularly in networks with low heterogeneity. This is most clearly the case when the standard deviation of the degree distribution is lower than a threshold somewhere around *σ* ≈ 6. When we express this heterogeneity in same way as we have for the empirical networks, we get *σ*^2^/*μ* = 7.2; a value that is larger than that found in any of the real networks.

From this we should expect that segregation, rather than heterogeneity, ought to be the principal reason why the performance of each strategy varies from one network to another. In fact, we do observe that modularity, here used as a measure of segregation, serves as a more reliable guide for choosing a strategy than heterogeneity; note that there are 4 networks in Fig 3 for which the global strategy is best at all sample sizes, and these are precisely the 4 for which modularity is lowest. Similarly, the component-based strategies perform best in those with highest modularity. While heterogeneity may have some effect, its influence is less apparent.

Finally, we have shown that the amount of data we have could also be informative in choosing the best strategy. Perhaps our most useful discovery is the result that the value of information decreases greatly as sample size increases, meaning that a 10% sample of the network can be almost as good as the whole thing. For networks that have a considerable amount of subdivision within the population, we have shown that the component-based strategies will perform better than the global strategy but only when the sample size is suitably small. Moreover, we can estimate by computing the left hand side of Eq (3), when this will be the case; a positive result suggests that the sample is larger than it needs to be to optimize the effectiveness of the component strategy (and possibly the proportional strategy).

While component based strategies can be worthwhile, it is usually the case that the most effective strategy is to simply target the highest degree individuals in the sample. This is more likely to be true when the node-sampling method is used such as when data was obtained through surveys of selected individuals (see [38] for an example). Link tracing and snowball sampling have also been found to be an effective way to obtain network contacts when privacy is an issue, as it would be in a community of drug users or a network of sexual relationships [39]. For this type of sampling component strategies would not be applicable as we would only have information from one component.

We have measured the success of each strategy by counting firstly, the number of cases that could potentially be prevented after detection; the advantage of this measure is that it is highest when (a) large outbreaks are detected, and (b) they are detected early; and secondly, the number of cases that occur before the disease becomes detected by a sentinel; this measure is most relevant to diseases that do not have extreme symptoms and can go undetected in the population for some time. Neither measure, however, takes into account the difficulties of actually preventing these cases. The method of prevention, for example vaccination, quarantine, or dissemination of information to the community, will take some amount of time and is unlikely to be 100% effective.

In some cases it may be possible to incorporate other sources of data. For example, we have assumed that all nodes are equally likely to be patient zero in our simulations whereas in many cases we might have a general idea about where the disease will originate from. For zoonotic disease like influenza it would be worth examining the benefits of placing sentinels close to farms or wildlife populations where such diseases are endemic. Questions also remain about whether our results apply to larger networks. In conclusion, the strategies and results we have presented here are not, in general, directly applicable real-world scenarios, but they may serve as guidelines for building more customized approaches.

We end this paper by commenting on the wider applicability of our results, and indeed all similar work in this area. Throughout the paper we have focused on the problem of infectious diseases, however, almost everything that has been said could equally be applied to several other contagion processes on networks such as computer viruses [40]. Another example is the spread of viral content on social media [41, 42]. Here the question is which online accounts should be monitored to predict the online trends of the future. Lastly, these analyses could be applied to the spread of information in criminal networks [43]. The objective here would be to intercept the communications of individuals who are likely to be involved in the diffusion of information about a planned drug deal or terrorist attack.

## Supporting information

S1 Fig Empirical degree distributions. The degree distributions for all the empirical networks.
(PDF)

S2 Fig Synthetic degree distributions. The degree distributions for 16 arbitrarily selected synthetic networks from the collection of randomly parameterized networks.
(PDF)

S3 Fig Outbreak size distributions. Outbreak size distributions for the single seed disease simulations for all the empirical networks.
(PDF)

S4 Fig Random strategies. Comparison of benchmark strategies. results are presented for the percentage of cases before detection in the multi-outbreak simulation with 5 sentinels.
(PDF)

S5 Fig CAD 3 sentinels node sampling. Cases after detection for the single seed simulation with 3 sentinels over a range of subsamples generated by sampling nodes in the network. Results are given as the mean percentage of the nodes in the outbreak infected after at least one sentinel was infected.
(PDF)

S6 Fig CAD 3 sentinels edge sampling. Cases after detection for the single seed simulation with 3 sentinels over a range of subsamples generated by sampling edges in the network. Results are given as the mean percentage of the nodes in the outbreak infected after at least one sentinel was infected.
(PDF)

S7 Fig CAD 5 sentinels node sampling. Cases after detection for the single seed simulation with 5 sentinels over a range of subsamples generated by sampling nodes in the network. Results are given as the mean percentage of the nodes in the outbreak infected after at least one sentinel was infected.
(PDF)

S8 Fig CAD 5 sentinels edge sampling. Cases after detection for the single seed simulation with 5 sentinels over a range of subsamples generated by sampling edges in the network. Results are given as the mean percentage of the nodes in the outbreak infected after at least one sentinel was infected.
(PDF)

S9 Fig CAD 10 sentinels node sampling. Cases after detection for the single seed simulation with 10 sentinels over a range of subsamples generated by sampling nodes in the network. Results are given as the mean percentage of the nodes in the outbreak infected after at least one sentinel was infected.
(PDF)

S10 Fig CAD 10 sentinels edge sampling. Cases after detection for the single seed simulation with 10 sentinels over a range of subsamples generated by sampling edges in the network. Results are given as the mean percentage of the nodes in the outbreak infected after at least one sentinel was infected.
(PDF)

S11 Fig CBD 3 sentinels node sampling. Cases before detection for the multiple seed simulation with 3 sentinels over a range of subsamples generated by sampling nodes in the network. Results are given as the mean percentage of the nodes in the network infected before at least one sentinel was infected.
(PDF)

S12 Fig CBD 3 sentinels edge sampling. Cases before detection for the multiple seed simulation with 3 sentinels over a range of subsamples generated by sampling edges in the network. Results are given as the mean percentage of the nodes in the network infected before at least one sentinel was infected.
(PDF)

S13 Fig CBD 5 sentinels node sampling. Cases before detection for the multiple seed simulation with 5 sentinels over a range of subsamples generated by sampling nodes in the network. Results are given as the mean percentage of the nodes in the network infected before at least one sentinel was infected.
(PDF)

S14 Fig CBD 5 sentinels edge sampling. Cases before detection for the multiple seed simulation with 5 sentinels over a range of subsamples generated by sampling edges in the network. Results are given as the mean percentage of the nodes in the network infected before at least one sentinel was infected.
(PDF)

S15 Fig CBD 10 sentinels node sampling. Cases before detection for the multiple seed simulation with 10 sentinels over a range of subsamples generated by sampling nodes in the network. Results are given as the mean percentage of the nodes in the network infected before at least one sentinel was infected.
(PDF)

S16 Fig CBD 10 sentinels edge sampling. Cases before detection for the multiple seed simulation with 10 sentinels over a range of subsamples generated by sampling edges in the network. Results are given as the mean percentage of the nodes in the network infected before at least one sentinel was infected.
(PDF)

S17 Fig CAD MR method. Difference in the number of cases after detection for sufficiently fragmented samples. Each marker represents one network. Empirical networks are represented by star shaped markers, synthetic networks are represented by circles. The edge sampling method was used and only samples that were evaluated to be sufficiently fragmented contribute to the results shown here. Red markers show where the global strategy performs better, i.e. prevents a larger number of cases, than the component strategy.
(PDF)

S18 Fig CBD MR method. Difference in the number of cases before detection for sufficiently fragmented samples. Each marker represents one network. Empirical networks are represented by star shaped markers, synthetic networks are represented by circles. The edge sampling method was used and only samples that were evaluated to be sufficiently fragmented contribute to the results shown here. Red markers show where the global strategy performs worse, i.e. yields a larger number of undetected cases, than the component strategy.
(PDF)
