# Supplementary figures and images for: Efficient sentinel surveillance strategies for preventing epidemics on networks
Source: PLoS Comput Biol. 2019 Nov 25;15(11):e1007517. doi: 10.1371/journal.pcbi.1007517 (PMC6910701; doi:10.1371/journal.pcbi.1007517)

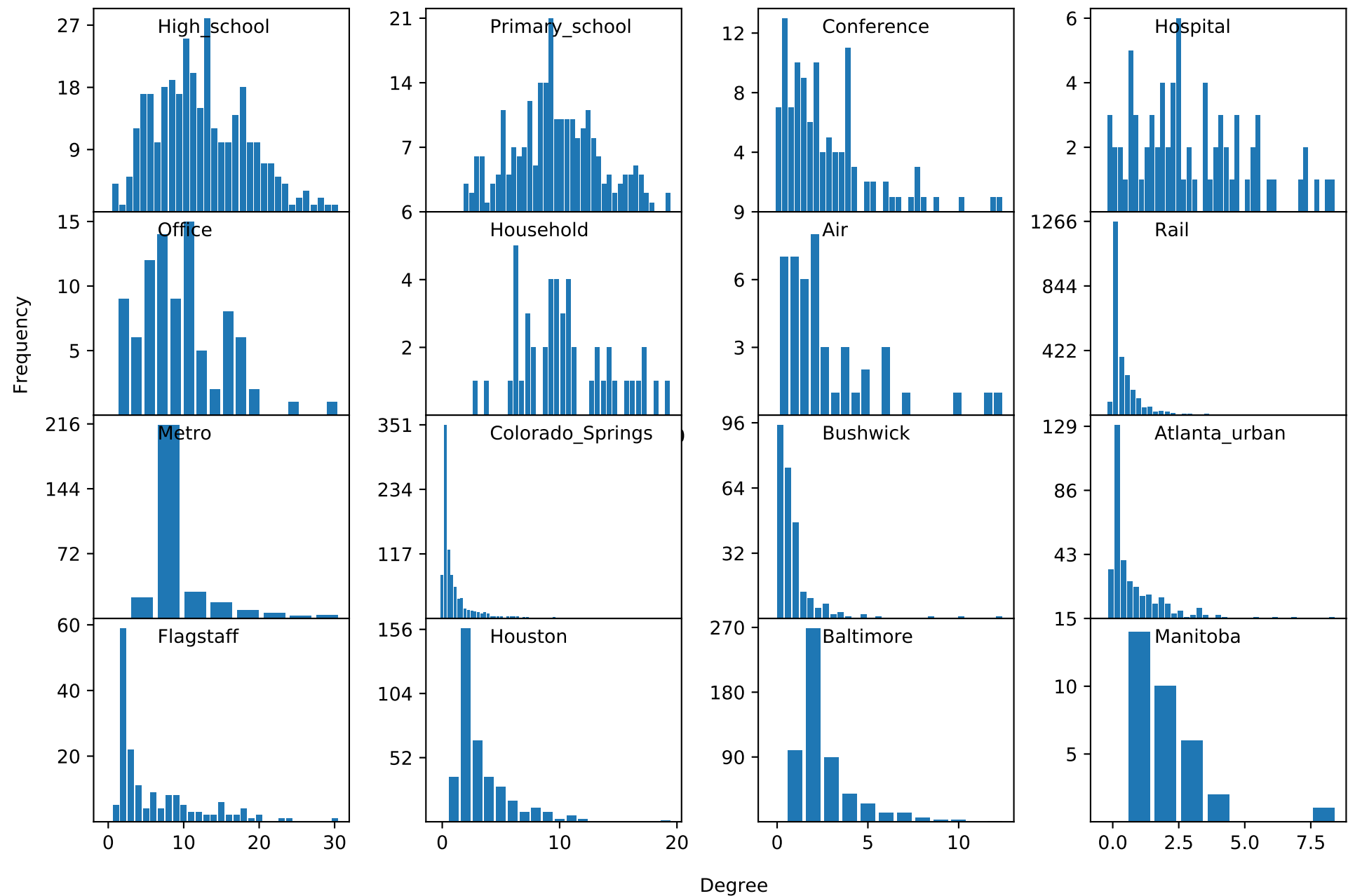

Supplement: S1 Fig — The degree distributions for all the empirical networks. (PDF) [file pcbi.1007517.s001.pdf]

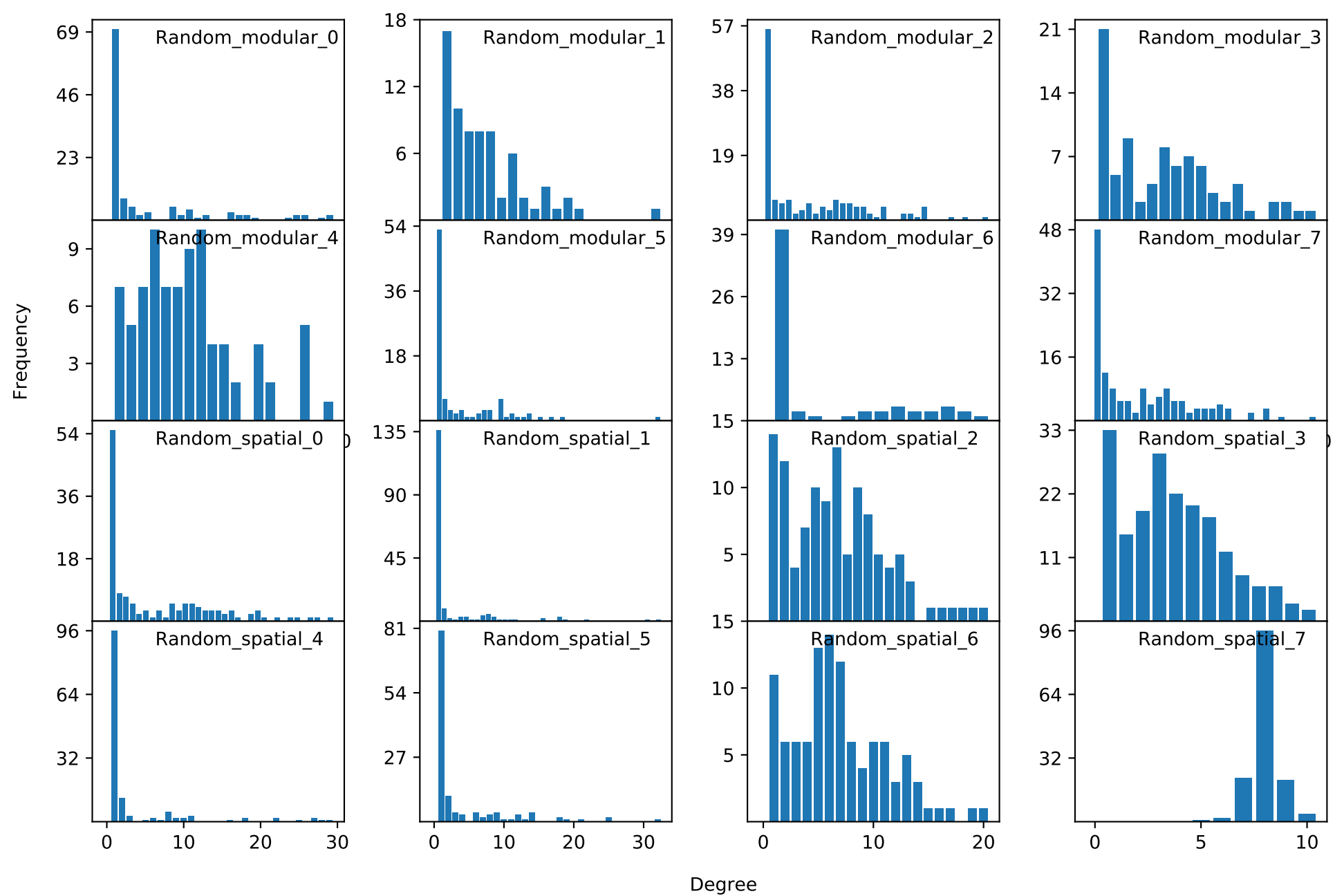

Supplement: S2 Fig — The degree distributions for 16 arbitrarily selected synthetic networks from the collection of randomly parameterized networks. (PDF) [file pcbi.1007517.s002.pdf]

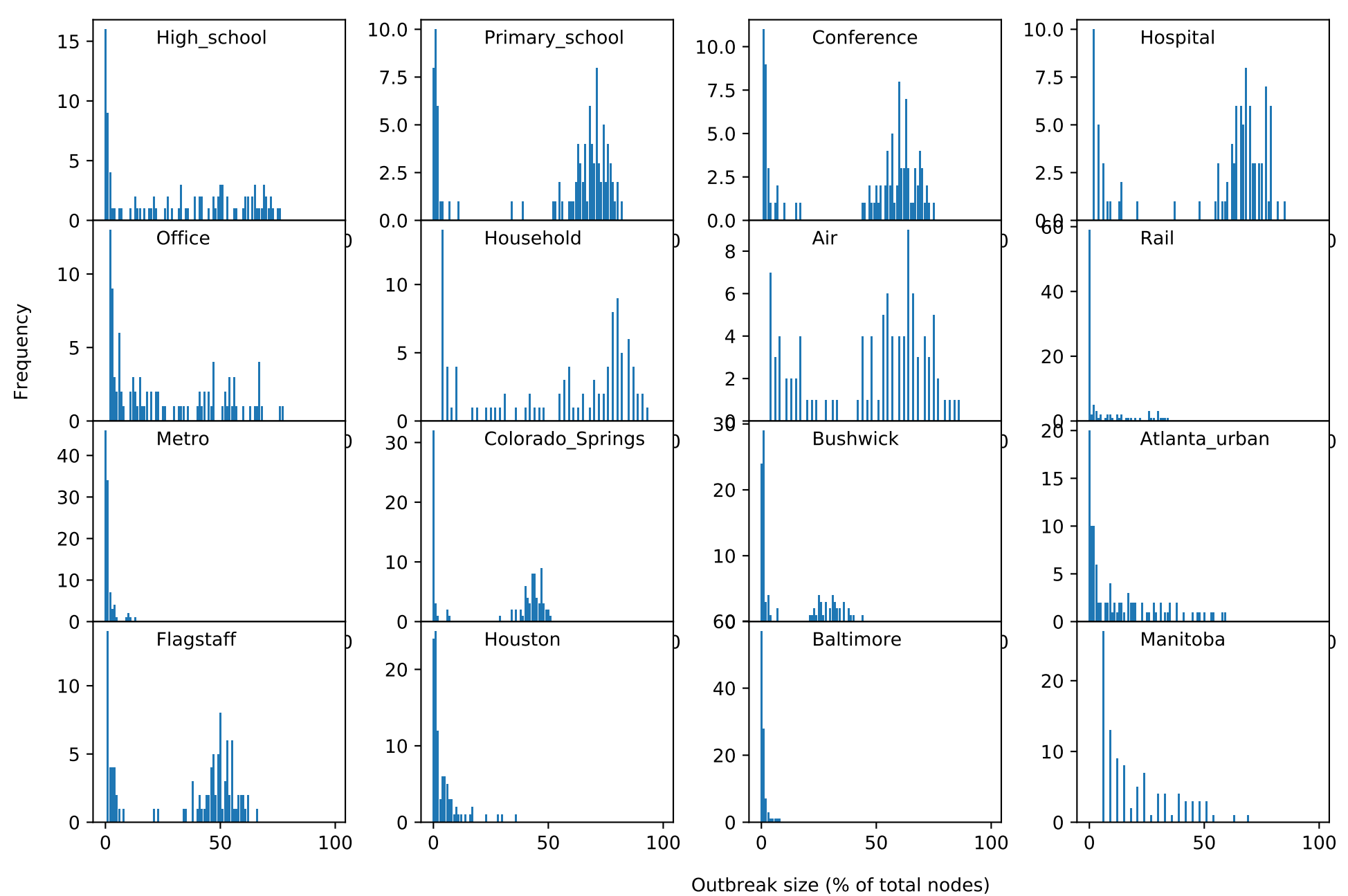

Supplement: S3 Fig — Outbreak size distributions for the single seed disease simulations for all the empirical networks. (PDF) [file pcbi.1007517.s003.pdf]

Global Acquaintance Random

% cases before detection (multiple seeds)

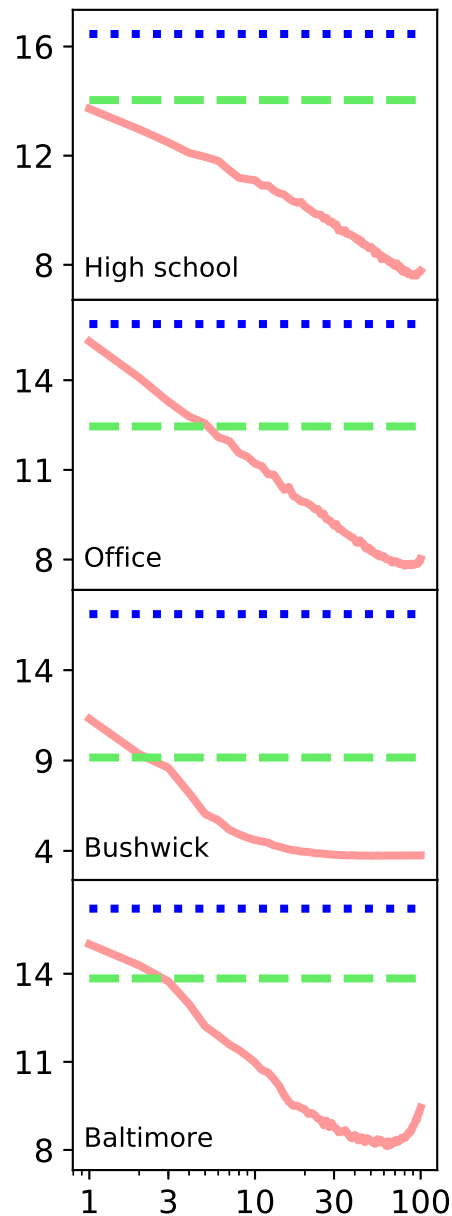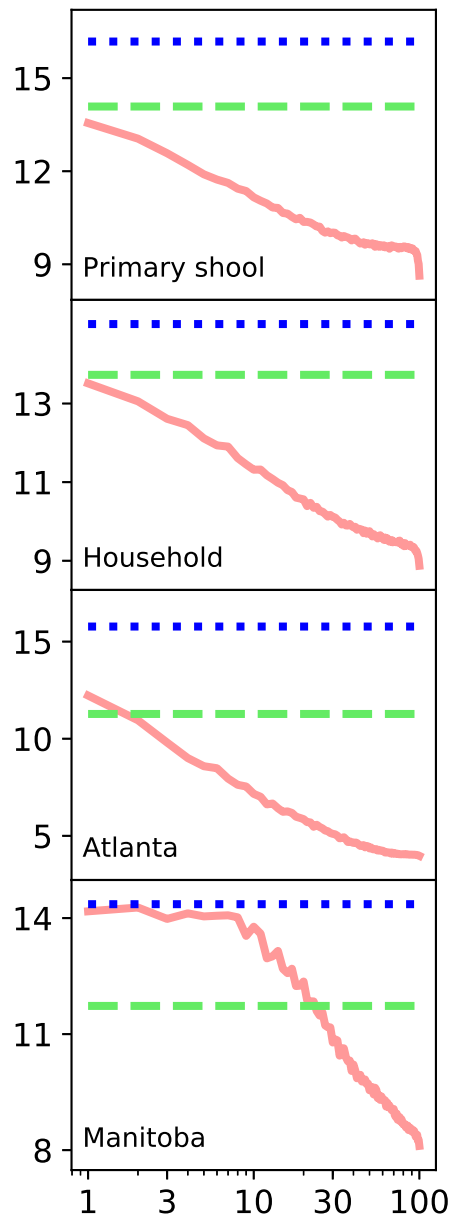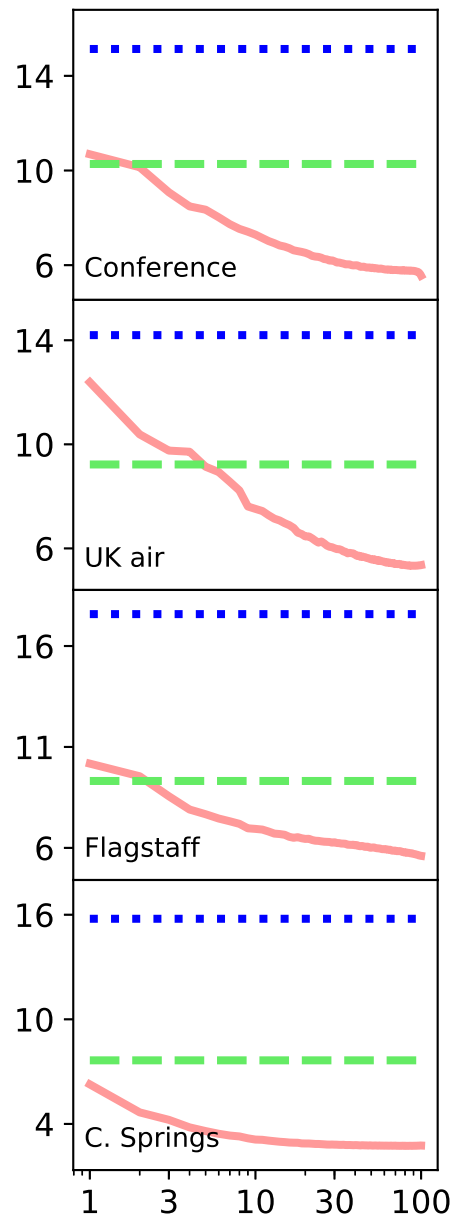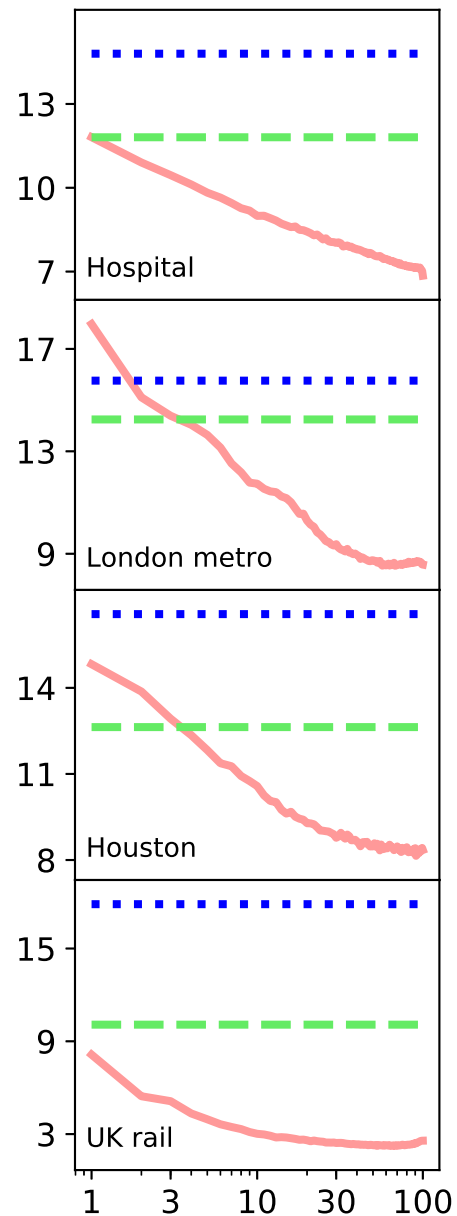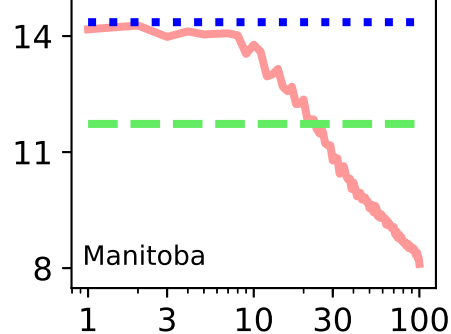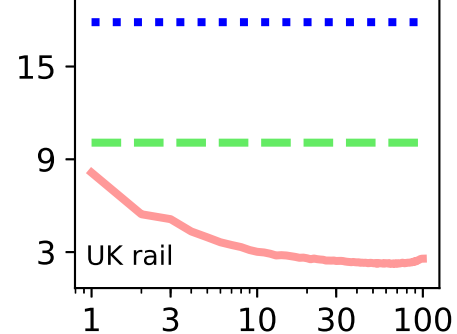

Sample size (% of total edges)

Supplement: S4 Fig — Comparison of benchmark strategies. results are presented for the percentage of cases before detection in the multi-outbreak simulation with 5 sentinels. (PDF) [file pcbi.1007517.s004.pdf]

Global Component Proportional

% cases after detection (single seed)

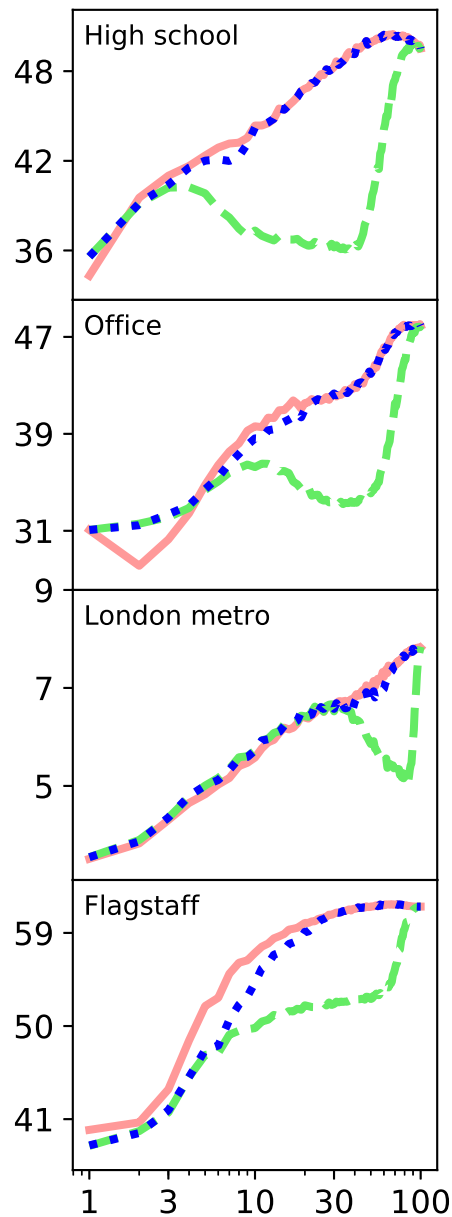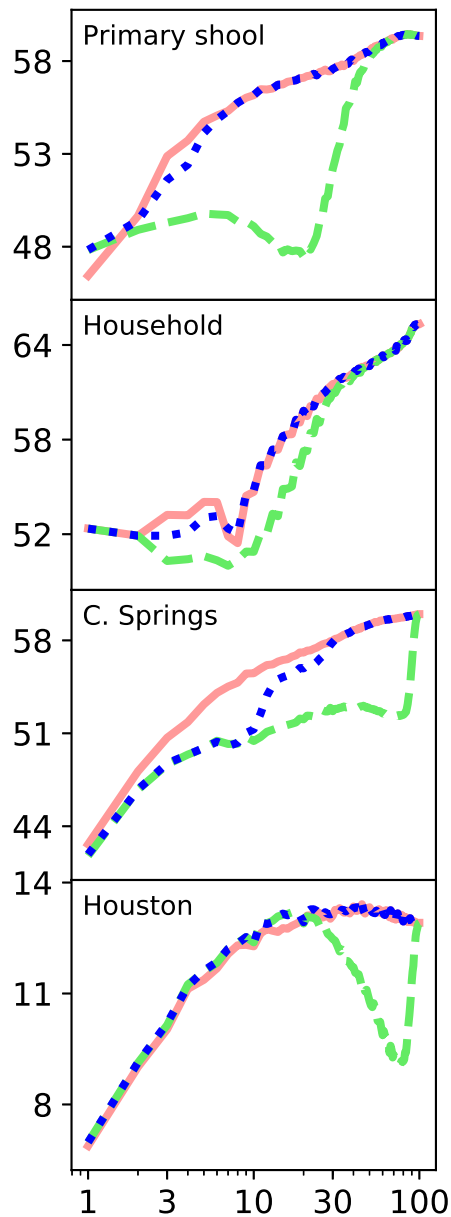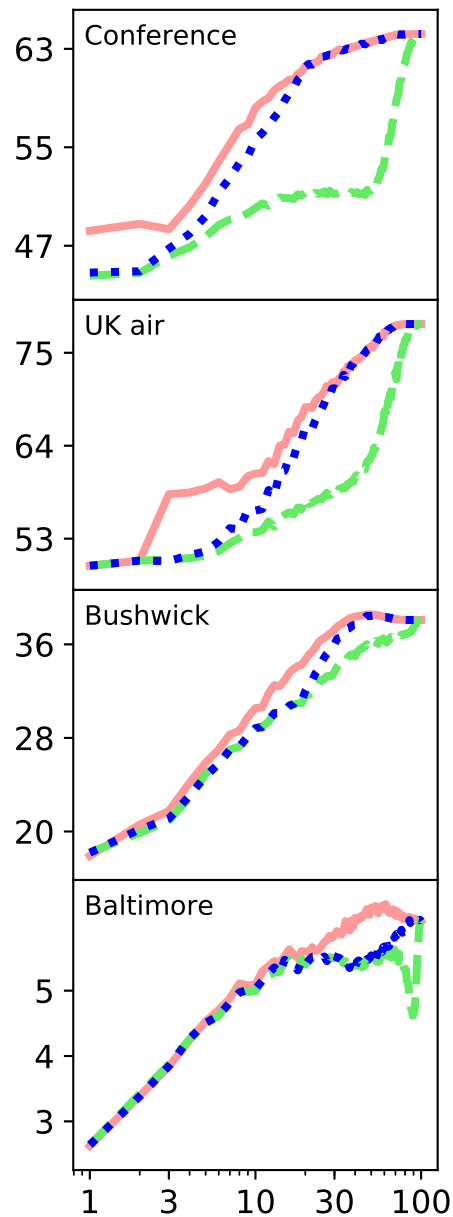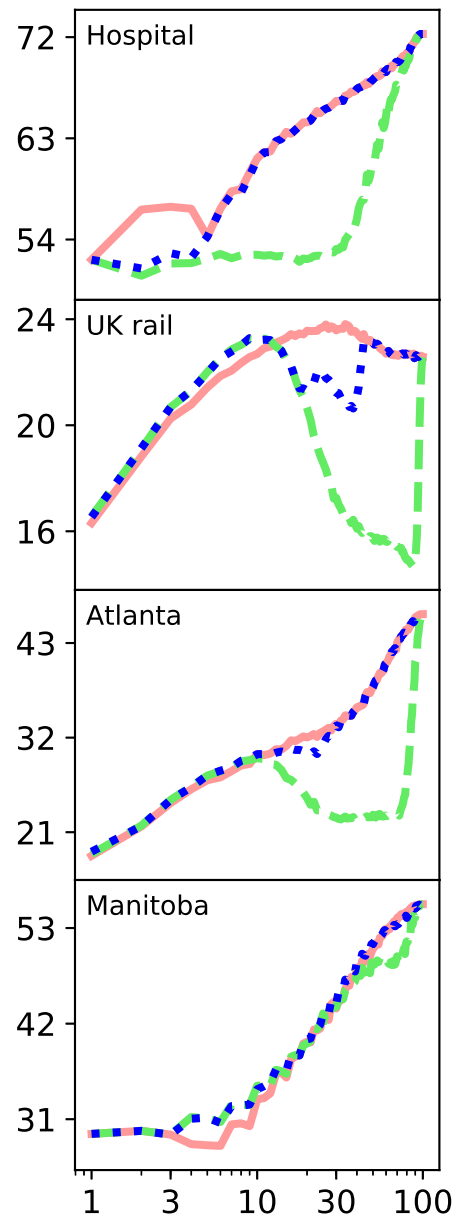

Sample size (% of total nodes)

Supplement: S5 Fig — Cases after detection for the single seed simulation with 3 sentinels over a range of subsamples generated by sampling nodes in the network. Results are given as the mean percentage of the nodes in the outbreak infected after at least one sentinel was infected. (PDF) [file pcbi.1007517.s005.pdf]

Global Component Proportional Molloy-Reed threshold

% cases after detection (single seed)

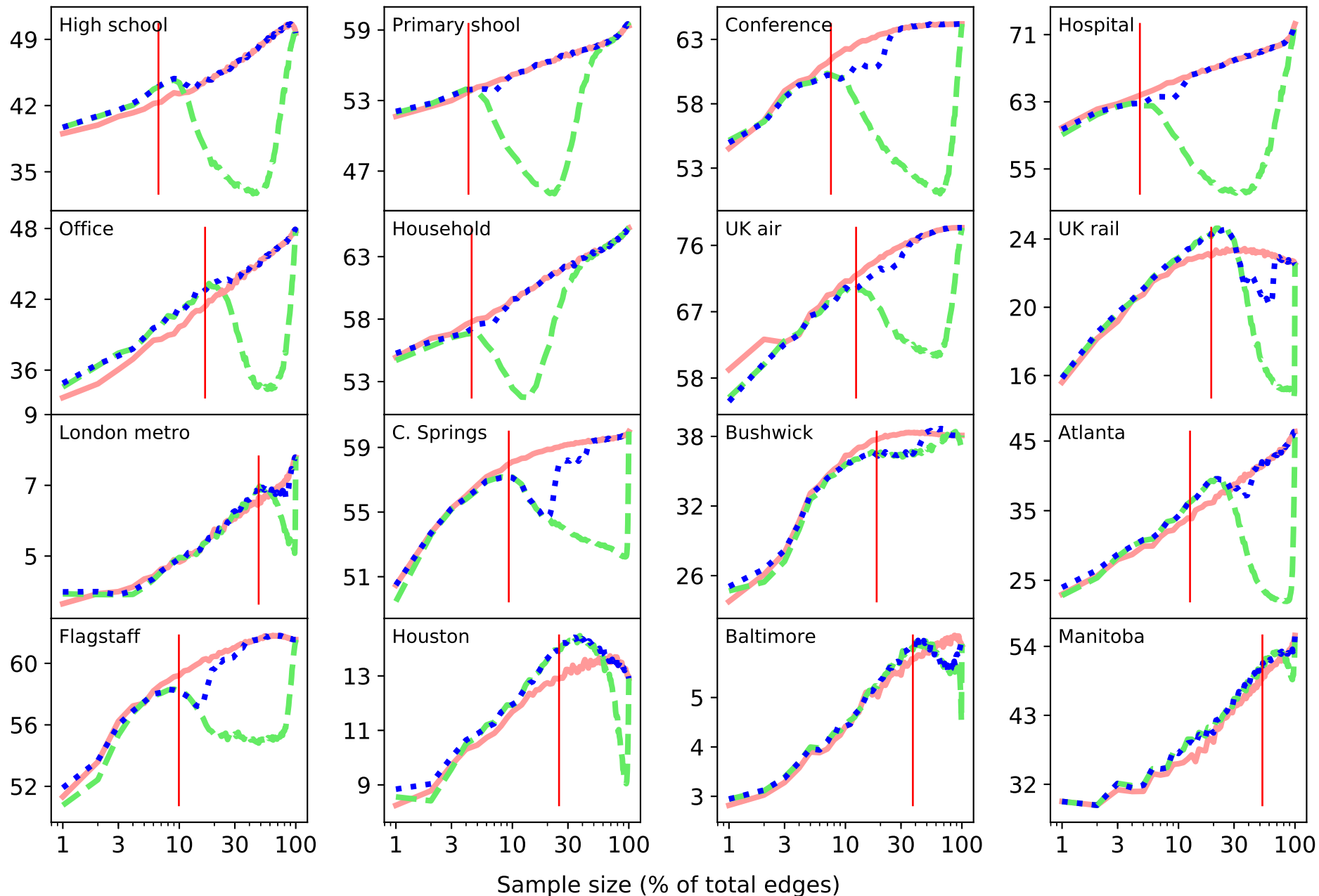

Supplement: S6 Fig — Cases after detection for the single seed simulation with 3 sentinels over a range of subsamples generated by sampling edges in the network. Results are given as the mean percentage of the nodes in the outbreak infected after at least one sentinel was infected. (PDF) [file pcbi.1007517.s006.pdf]

Global Component Proportional

% cases after detection (single seed)

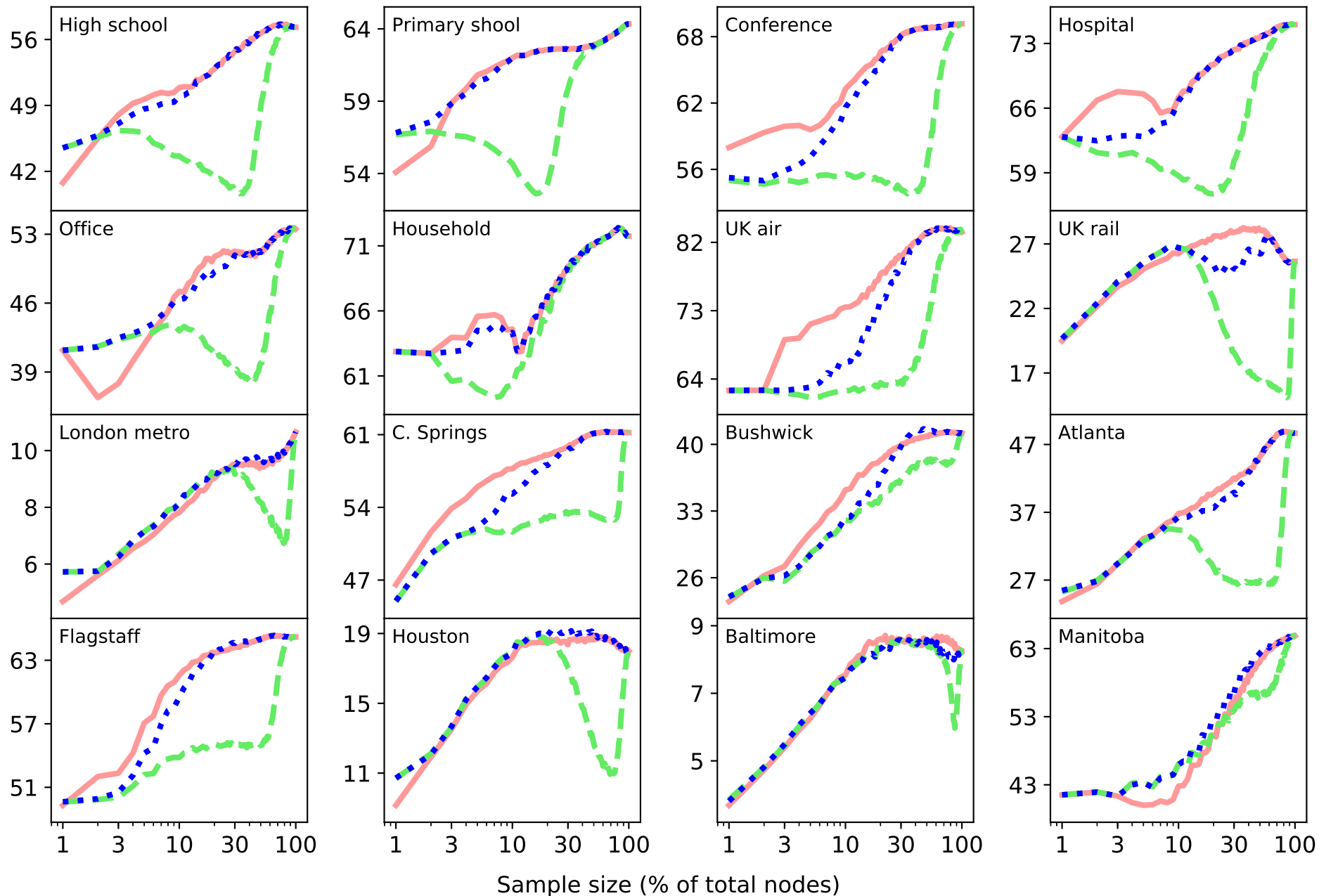

Supplement: S7 Fig — Cases after detection for the single seed simulation with 5 sentinels over a range of subsamples generated by sampling nodes in the network. Results are given as the mean percentage of the nodes in the outbreak infected after at least one sentinel was infected. (PDF) [file pcbi.1007517.s007.pdf]

Global    Component    Proportional    Molloy-Reed threshold

% cases after detection (single seed)

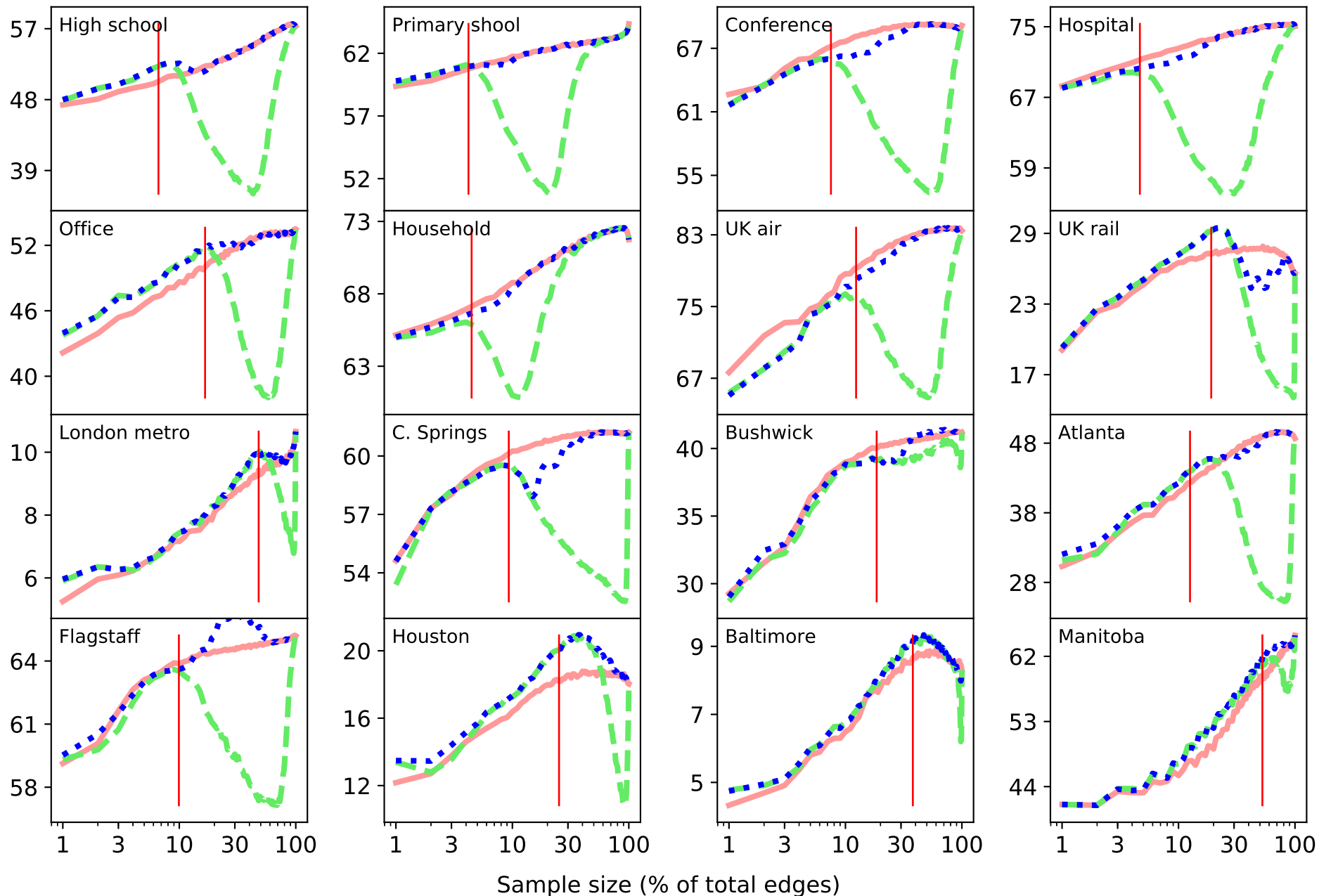

Supplement: S8 Fig — Cases after detection for the single seed simulation with 5 sentinels over a range of subsamples generated by sampling edges in the network. Results are given as the mean percentage of the nodes in the outbreak infected after at least one sentinel was infected. (PDF) [file pcbi.1007517.s008.pdf]

Global Component Proportional

% cases after detection (single seed)

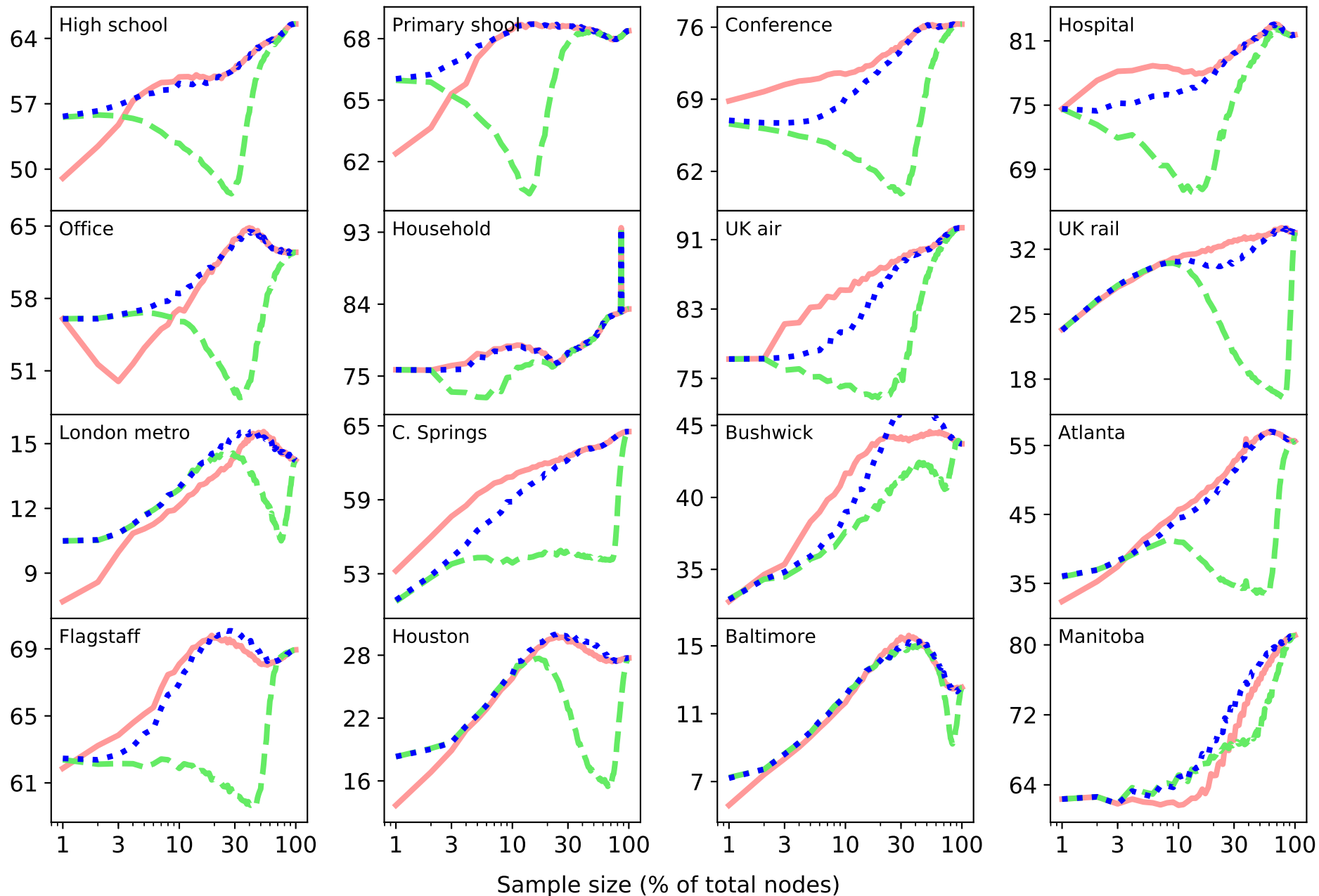

Supplement: S9 Fig — Cases after detection for the single seed simulation with 10 sentinels over a range of subsamples generated by sampling nodes in the network. Results are given as the mean percentage of the nodes in the outbreak infected after at least one sentinel was infected. (PDF) [file pcbi.1007517.s009.pdf]

Global Component Proportional Molloy-Reed threshold

% cases after detection (single seed)

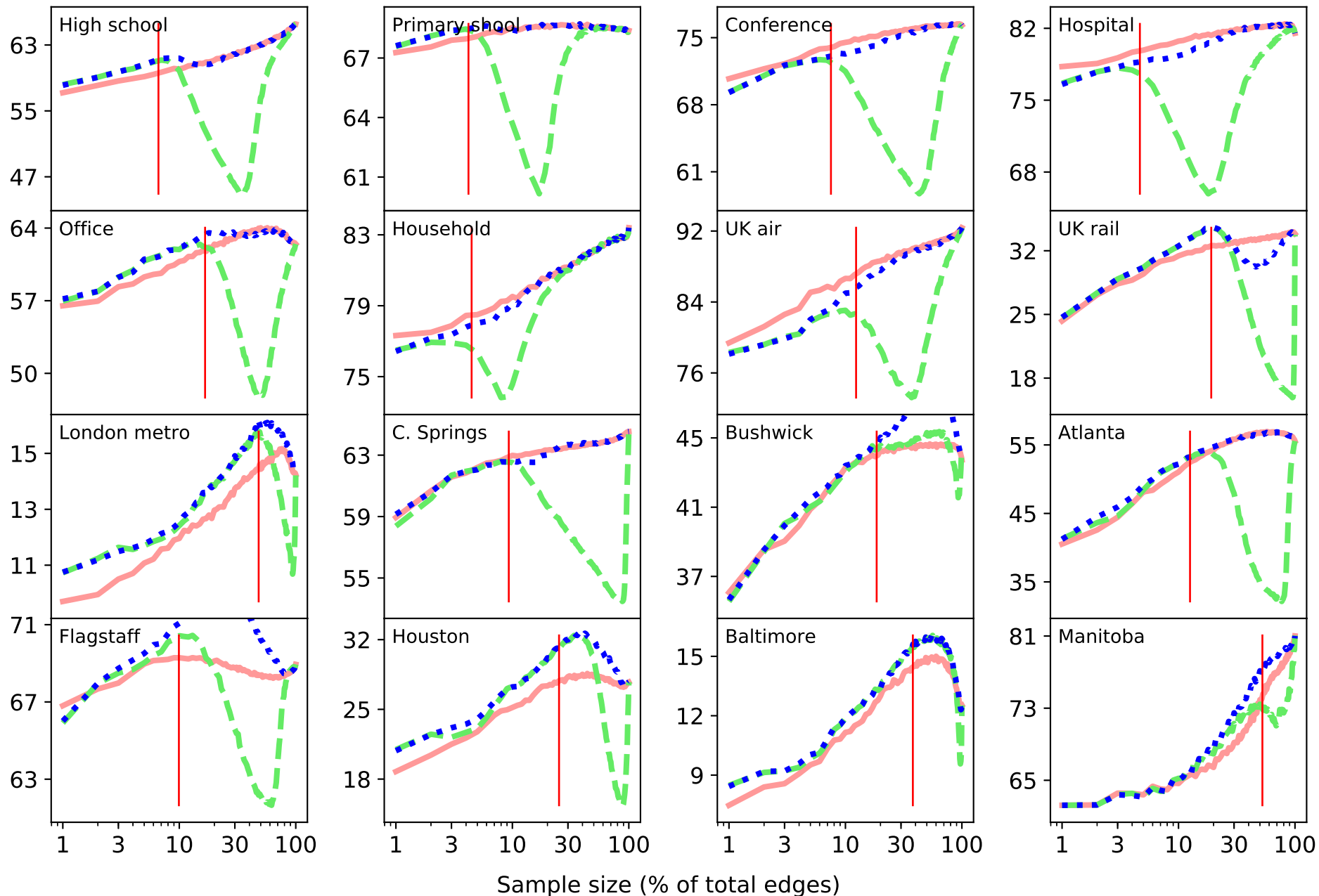

Supplement: S10 Fig — Cases after detection for the single seed simulation with 10 sentinels over a range of subsamples generated by sampling edges in the network. Results are given as the mean percentage of the nodes in the outbreak infected after at least one sentinel was infected. (PDF) [file pcbi.1007517.s010.pdf]

Global Component Proportional

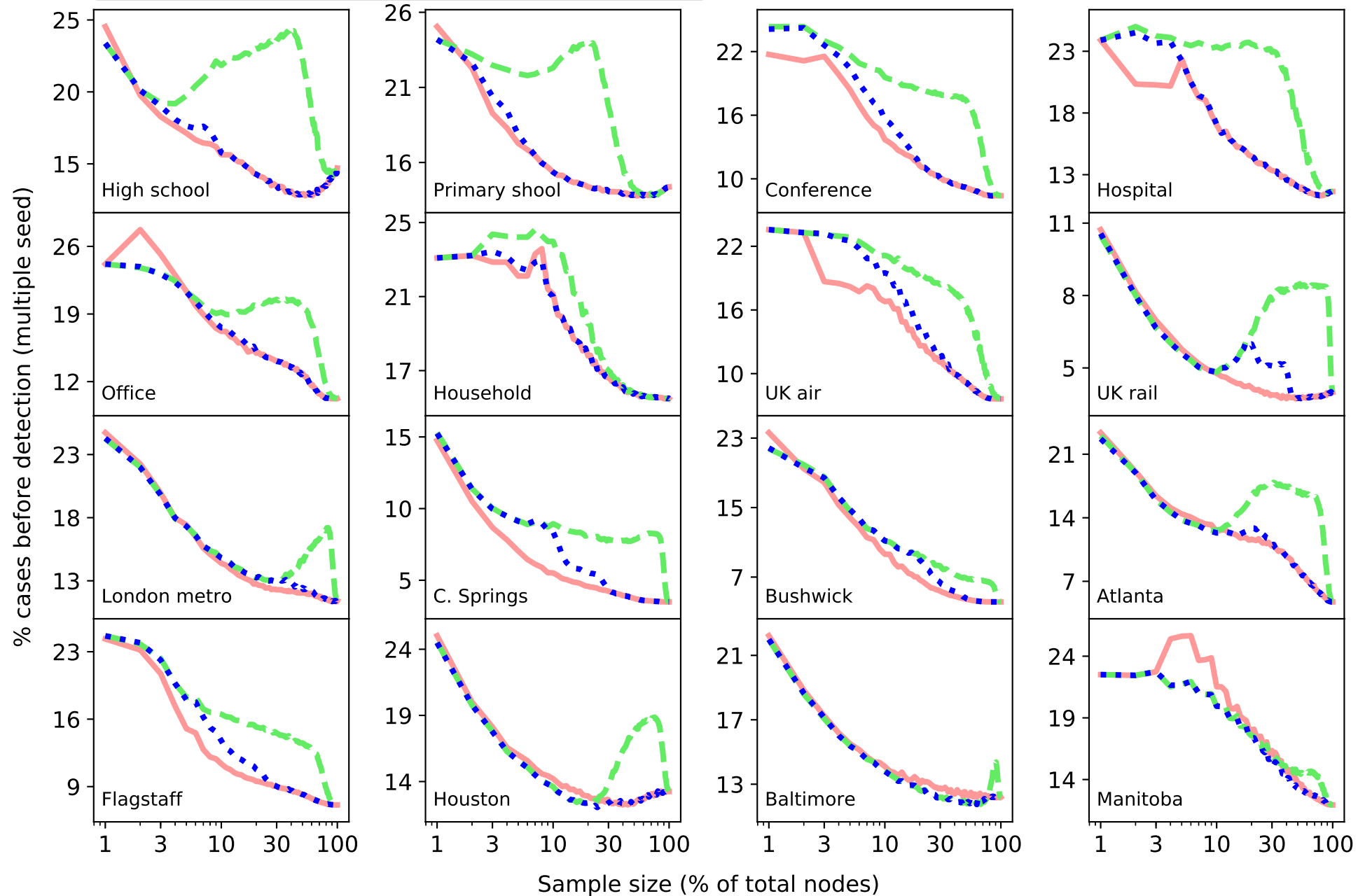

Supplement: S11 Fig — Cases before detection for the multiple seed simulation with 3 sentinels over a range of subsamples generated by sampling nodes in the network. Results are given as the mean percentage of the nodes in the network infected before at least one sentinel was infected. (PDF) [file pcbi.1007517.s011.pdf]

Global Component Proportional Molloy-Reed threshold

% cases before detection (multiple seed)

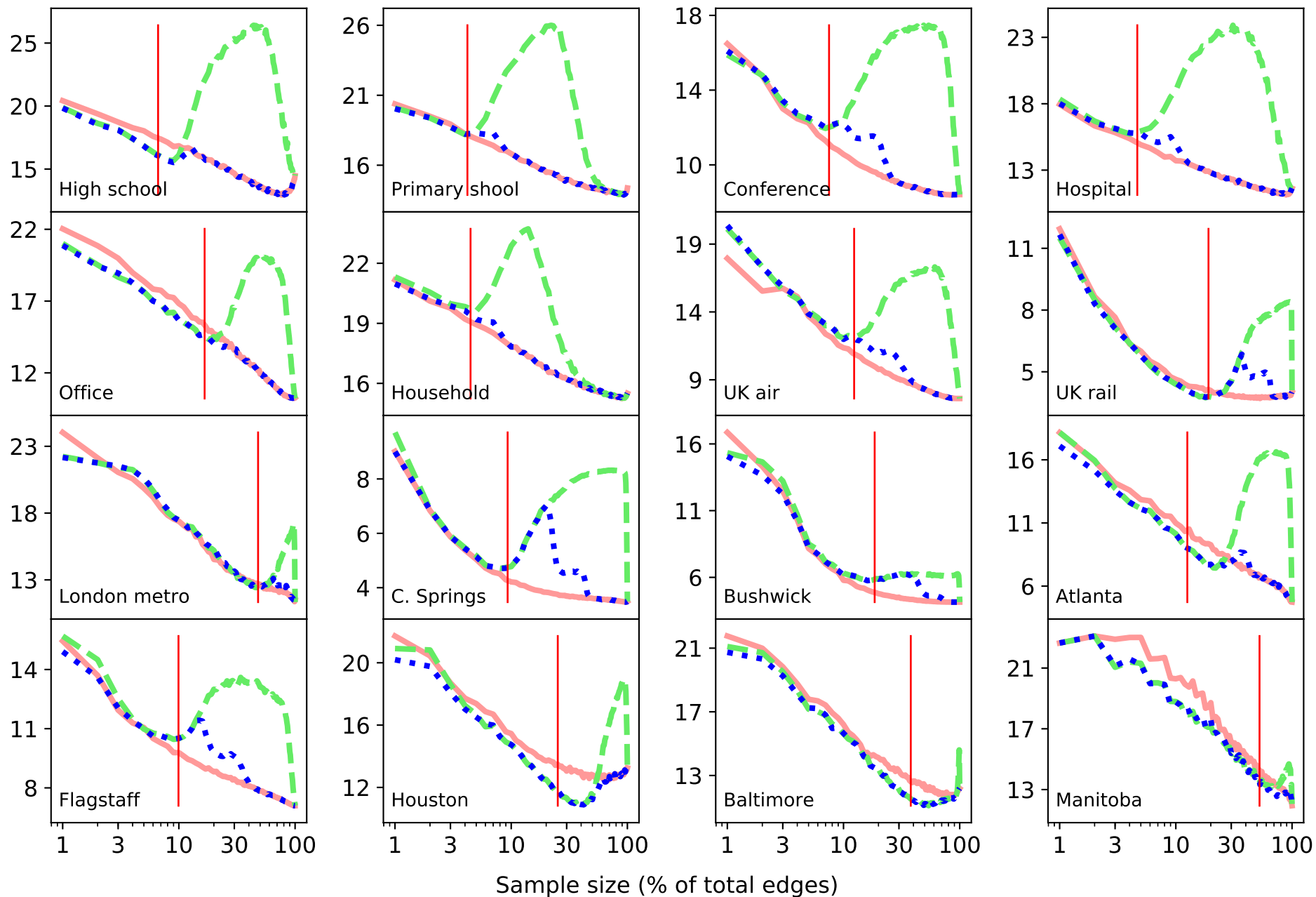

Supplement: S12 Fig — Cases before detection for the multiple seed simulation with 3 sentinels over a range of subsamples generated by sampling edges in the network. Results are given as the mean percentage of the nodes in the network infected before at least one sentinel was infected. (PDF) [file pcbi.1007517.s012.pdf]

Global Component Proportional Molloy-Reed threshold

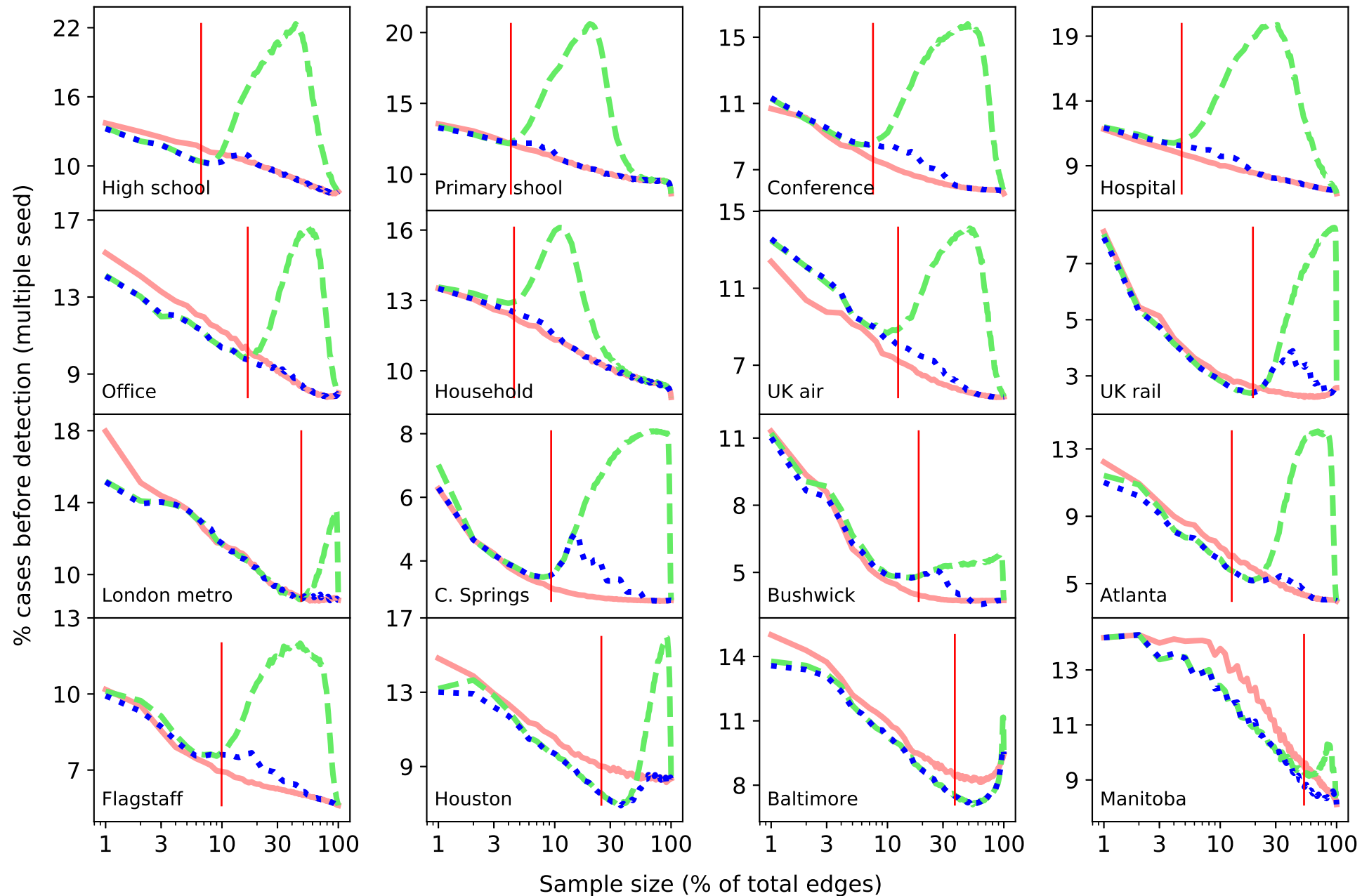

Supplement: S14 Fig — Cases before detection for the multiple seed simulation with 5 sentinels over a range of subsamples generated by sampling edges in the network. Results are given as the mean percentage of the nodes in the network infected before at least one sentinel was infected. (PDF) [file pcbi.1007517.s014.pdf]

Global    Component    Proportional    Molloy-Reed threshold

% cases before detection (multiple seed)

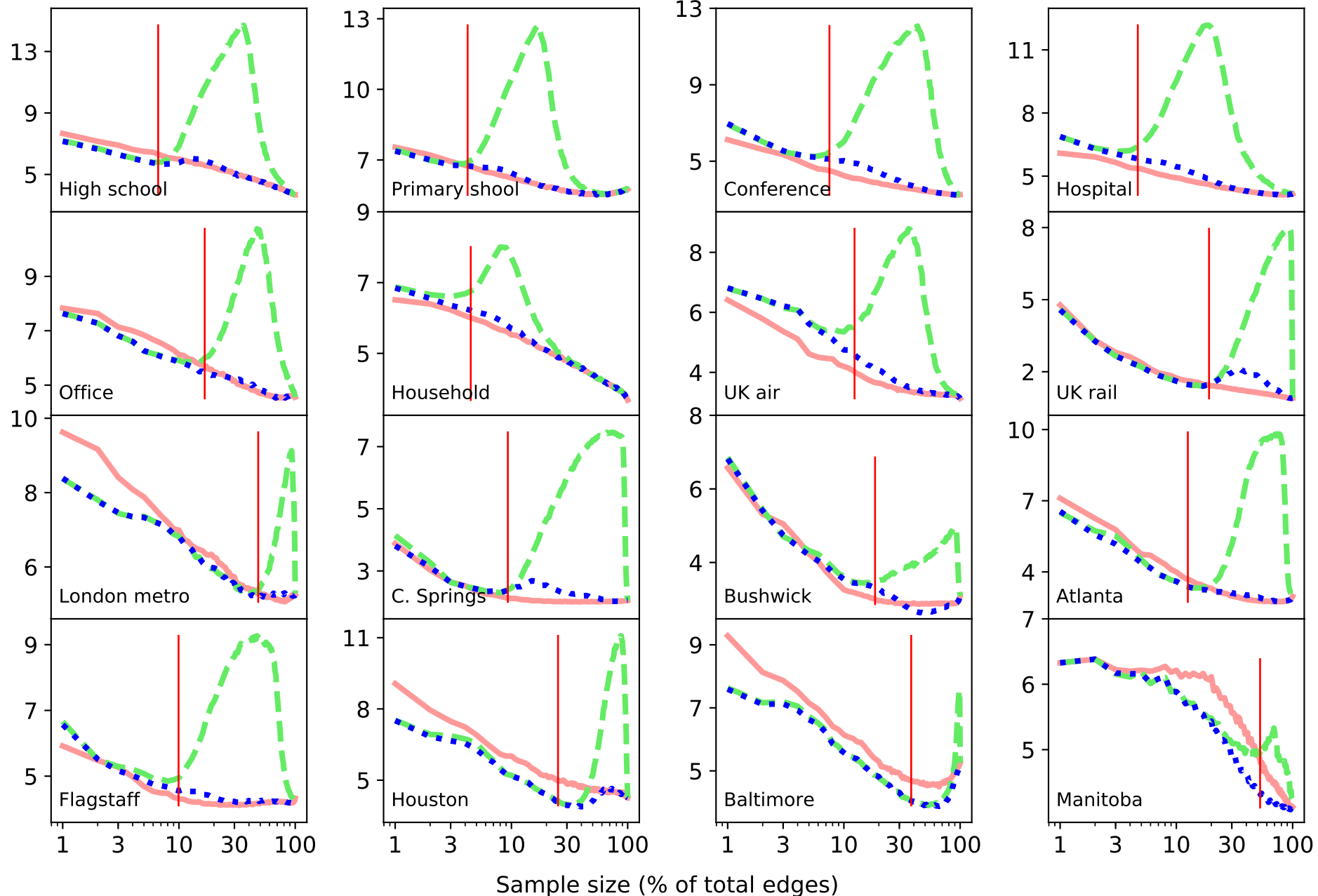

Supplement: S16 Fig — Cases before detection for the multiple seed simulation with 10 sentinels over a range of subsamples generated by sampling edges in the network. Results are given as the mean percentage of the nodes in the network infected before at least one sentinel was infected. (PDF) [file pcbi.1007517.s016.pdf]

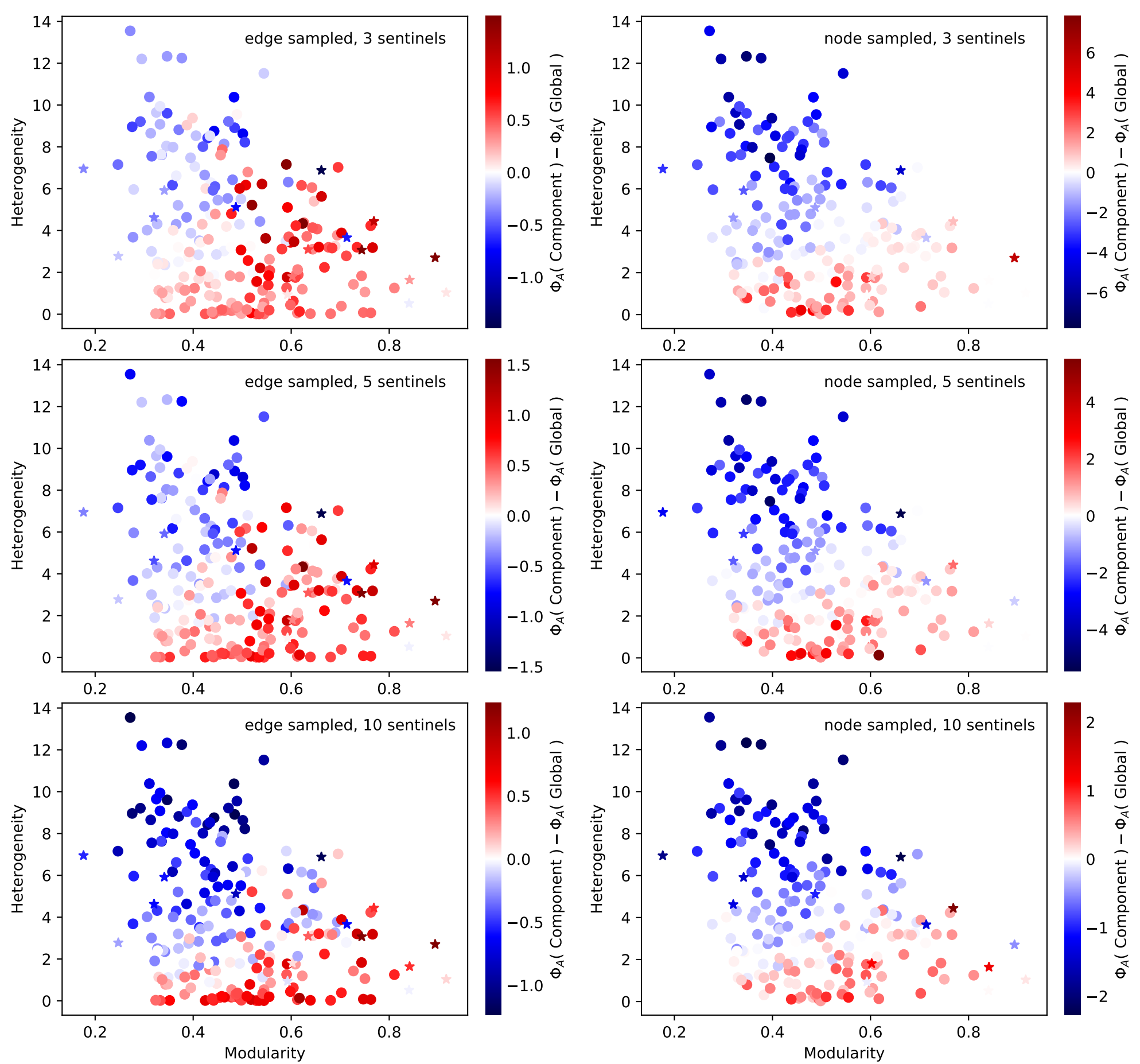

Supplement: S17 Fig — Difference in the number of cases after detection for sufficiently fragmented samples. Each marker represents one network. Empirical networks are represented by star shaped markers, synthetic networks are represented by circles. The edge sampling method was used and only samples that were evaluated to be sufficiently fragmented contribute to the results shown here. Red markers show where the global strategy performs better, i.e. prevents a larger number of cases, than the component strategy. (PDF) [file pcbi.1007517.s017.pdf]

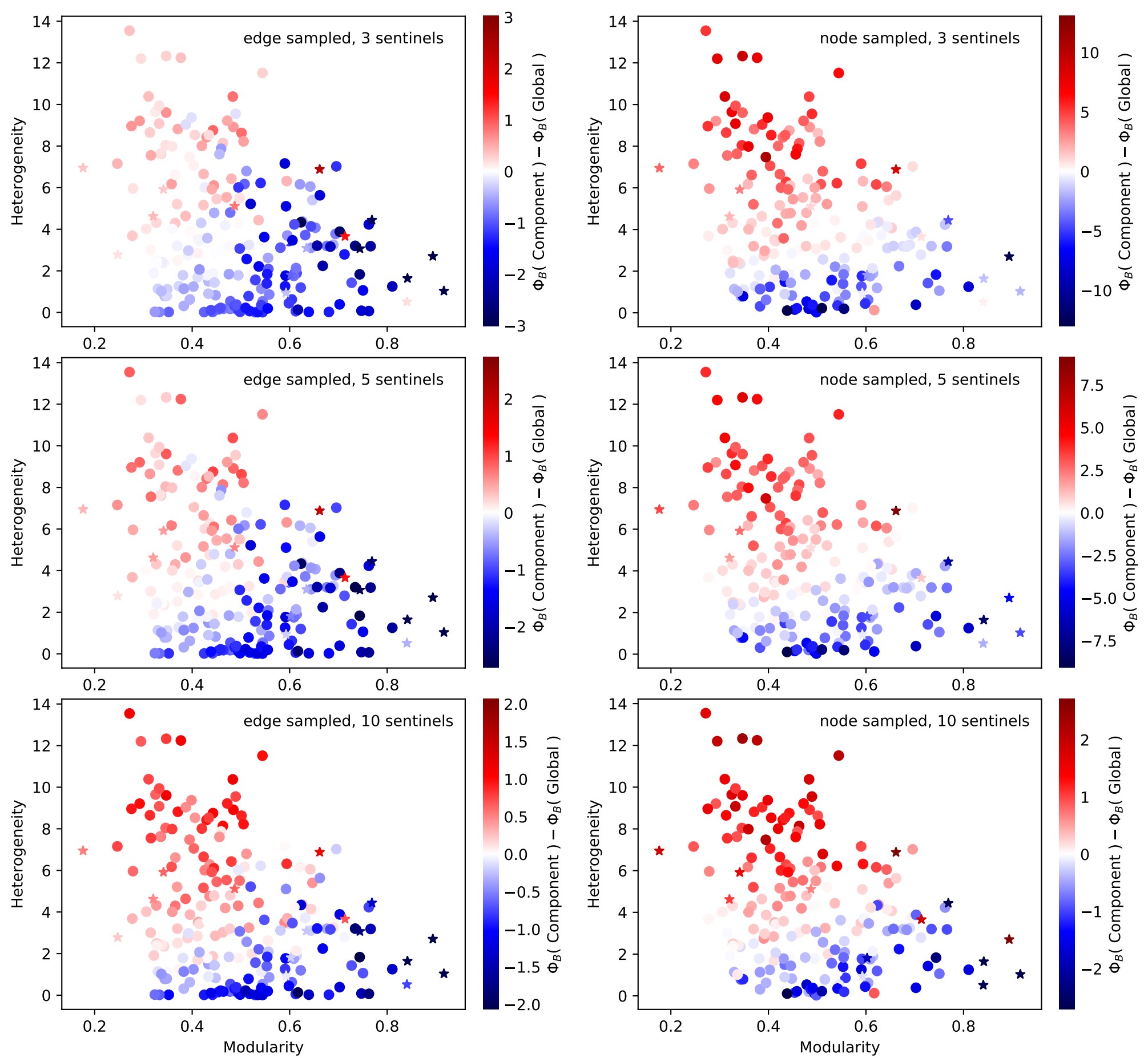

Supplement: S18 Fig — Difference in the number of cases before detection for sufficiently fragmented samples. Each marker represents one network. Empirical networks are represented by star shaped markers, synthetic networks are represented by circles. The edge sampling method was used and only samples that were evaluated to be sufficiently fragmented contribute to the results shown here. Red markers show where the global strategy performs worse, i.e. yields a larger number of undetected cases, than the component strategy. (PDF) [file pcbi.1007517.s018.pdf]
