# Supplementary material for: Efficient sentinel surveillance strategies for preventing epidemics on networks
Source: PLoS Comput Biol. 2019 Nov 25;15(11):e1007517. doi: 10.1371/journal.pcbi.1007517 (PMC6910701; doi:10.1371/journal.pcbi.1007517)
Supplement: S15 Fig — Cases before detection for the multiple seed simulation with 10 sentinels over a range of subsamples generated by sampling nodes in the network. Results are given as the mean percentage of the nodes in the network infected before at least one sentinel was infected. (PDF) [file pcbi.1007517.s015.pdf]

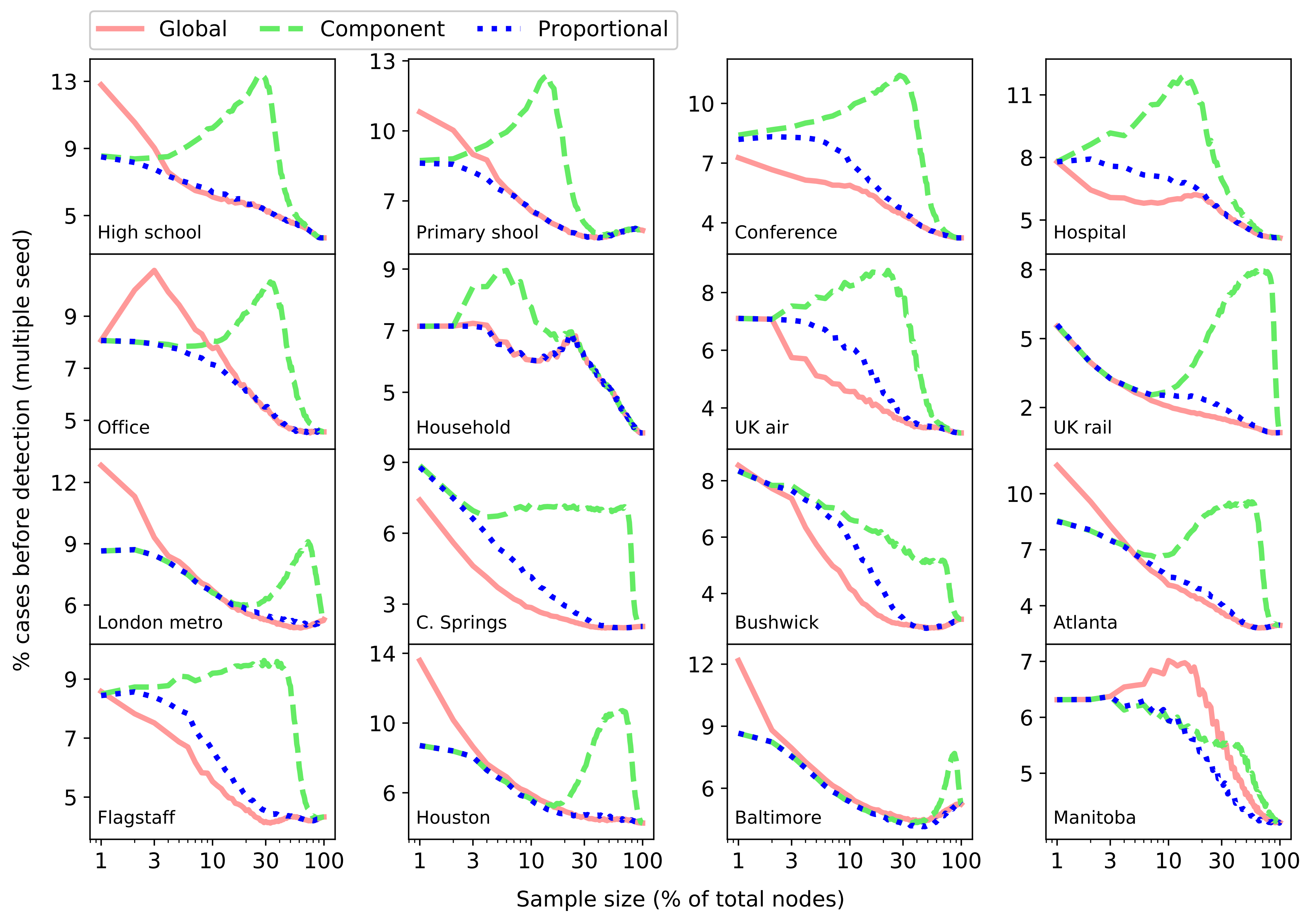

Global
Component
Proportional
% cases before detection (multiple seed)
Sample size (% of total nodes)
High school
Primary shool
Conference
Hospital
Office
Household
UK air
UK rail
London metro
C. Springs
Bushwick
Atlanta
Flagstaff
Houston
Baltimore
Manitoba
1
3
10
30
100
